# Healthcare workers’ perceptions of health worker-related interventions to improve compliance with hand hygiene recommendations for infection prevention and control in hospitalised neonates and infants in Sub-Saharan Africa: a synthesis of two qualitative evidence syntheses

**DOI:** 10.1080/16549716.2026.2621448

**Published:** 2026-02-17

**Authors:** Elodie Besnier, Dachi Arikpo, Deborah Ndukwu, Emmanuel Effa, Simon Lewin

**Affiliations:** a Department of Health Sciences in Ålesund, Norwegian University of Science and Technology, Ålesund, Norway; b Cochrane Nigeria, Institute of Tropical Diseases Research and Prevention, University of Calabar Teaching Hospital, Calabar, Nigeria; c Department of Internal Medicine, Faculty of Clinical Sciences, University of Calabar, Calabar, Nigeria; d Health Systems Research Unit, South African Medical Research Council, Cape Town, South Africa; e Centre for Epidemic Interventions Research, Norwegian Institute of Public Health, Oslo, Norway

**Keywords:** healthcare worker, qualitative evidence synthesis, hand hygiene, Sub-Saharan Africa, systematic review, hand hygiene compliance, acceptability, feasibility, implementation

## Abstract

Hand hygiene (HH) reduces infections in hospitalised neonates and infants. The benefits of HH may be compromised by poor compliance by healthcare workers (HCW). We carried out a synthesis of two qualitative evidence syntheses (QES) to explore HCWs’ perceptions of interventions to improve HH compliance (HHC) by health workers to prevent infections in hospitalised neonates and infants in Sub-Saharan Africa. We identified one existing QES – Chatfield 2017 – but identified gaps related to settings, scope and date of last search. To address these gaps, we carried out a new QES focused on studies from secondary and tertiary hospitals in Sub-Saharan Africa, published since 2015. We searched CINAHL, Embase, PubMed and African Journals Online. We carried out a thematic analysis and applied GRADE-CERQual to assess our confidence in the findings. We thereafter developed a novel approach to synthesise the findings of the two QES, using the qualitative evidence domains of the GRADE Evidence-to-decision framework. Finally, we reassessed our confidence GRADEings for each synthesised finding. Our synthesis of QES encompasses 37 publications (35 from Chatfield and 2 from our QES). It highlights that HH training and education, and reminders and communication interventions are acceptable to HCWs. However, they feel that the content, scope and/or target audience of these interventions should be enhanced to improve HHC (e.g. to include all staff and practical training). Findings on the acceptability of audit and feedback interventions are mixed, suggesting variations across settings. Our synthesis also highlights key institutional and environmental factors that can enhance HHC interventions.

## Paper Context

- **Main findings:** Hand hygiene compliance training and reminders in hospitals seem acceptable to healthcare workers, although institutional and environmental barriers may affect their feasibility locally and there is limited evidence from Sub-Saharan Africa.
- **Added knowledge:** This synthesis provides up-to-date evidence on the acceptability, feasibility, equity impacts of hand hygiene compliance interventions in Sub-Saharan African hospitals and on considerations for their implementation.
- **Global health impact for policy and action:** The synthesis findings have been used to inform the development of national guidelines in Nigeria. They may also be useful for informing hand hygiene compliance intervention guidelines and implementation in other countries of the region.

## Background

Healthcare-associated (nosocomial) infections (HAIs) are infections that begin ≥48 hours after hospitalisation or within 30 days after receiving healthcare [1]. HAIs are a common problem for premature infants and term infants with medical disorders requiring prolonged hospitalisation in intensive care units (NICUs/PICUs) [2]. Hand Hygiene (HH), ‘a way of cleaning one’s hands that substantially reduces potential pathogens (harmful microorganisms) on the hands’ [3], is effective in reducing HAIs in hospitalised neonates and infants [4]. The World Health Organization (WHO) advocates that HH should be performed by healthcare workers (HCW) and carers, before touching hospital equipment and instruments, before handling neonates, and between cleaning and caring for neonates [5]. The benefits of HH may not, however, be realised where there is poor hand hygiene compliance (HHC) by HCWs. A systematic review found an average HHC rate of 41.2% among HCWs in PICUs [6].

The WHO research agenda [7] for HH comprises six key domains for HHC improvement interventions (Box 1). Three of these domains – Training and education; Evaluation and feedback; and Reminders and communication – are primarily HCW-directed (individual interventions), while the other domains are primarily directed at the health system (organisational interventions). This QES focuses on HCW-directed HHC interventions.

**Box 1.** WHO’s key domains for HHC improvement interventions.

1. System Change: Interventions or approaches to facilitate changing systems to perform HH 2. Training and education: hand hygiene training, educational strategies on knowledge and skills needed to improve HH 3. Evaluation and feedback: monitoring of HH indicators, audits, data use to inform decision and influence behaviours 4. Reminders and communication: HH communication and reminder strategies towards HCW 5. Institutional safety climate: organisational factor supporting the institutionalisation of HH 6. Impact of HH improvement on HAIs and antimicrobial resistance (AMR): Impact of HHC and HAI/AMR prevalence

Most HHC interventions involve improving HCWs’ knowledge/self-efficacy, removing perceived or actual barriers to HH practice or providing cues for HH procedures [8]. Therefore, they help to facilitate the performance of and compliance with HH. Although HH is a simple and effective intervention to prevent HAIs, the quality and consistency of HH practices are low in many low- and middle-income countries and vary across settings. A 2019 systematic review on HHC in intensive care units found HHC below 50% in middle-income countries and as low as 9% in low-income countries [6]. The COVID-19 pandemic also highlighted the importance of HH in preventing the spread of infectious diseases and led to changes in HH practices and guidelines. However, research on HHC and HH interventions from the African region remains limited [6,9]. Understanding the key factors affecting the feasibility, acceptability and equity implications of various HHC interventions is essential for informing clinical guidelines relevant to the Sub-Saharan African context, to guide HH practice and develop HHC implementation strategies.

### Objectives

The aim of this synthesis of qualitative evidence syntheses (QES) is to explore HCWs perceptions (including healthcare professionals and support staff working in hospitals) of training and education; reminders and communication; and audit and feedback interventions to improve HCWs’ compliance with HH recommendations for infection prevention and control (IPC) in hospitalised neonates and infants in Sub-Saharan Africa (SSA).

This synthesis was developed to inform a national guideline development process in Nigeria [10], as part of the Global Evidence – Local Adaptation (GELA) project [11]. Therefore, we framed our research questions according to the GRADE evidence-to-decision (EtD) framework that we planned to use to structure the evidence presented to the Guideline Development Group [12]:

– How do HCWs in hospital settings perceive the feasibility of these interventions?
– How do HCWs in hospital settings perceive the acceptability of these interventions?
– How do HCWs in hospital settings perceive the impacts of these interventions on equity?
– What do HCWs in hospital settings see as important implementation considerations for these interventions?

## Method

**Figure 1.**
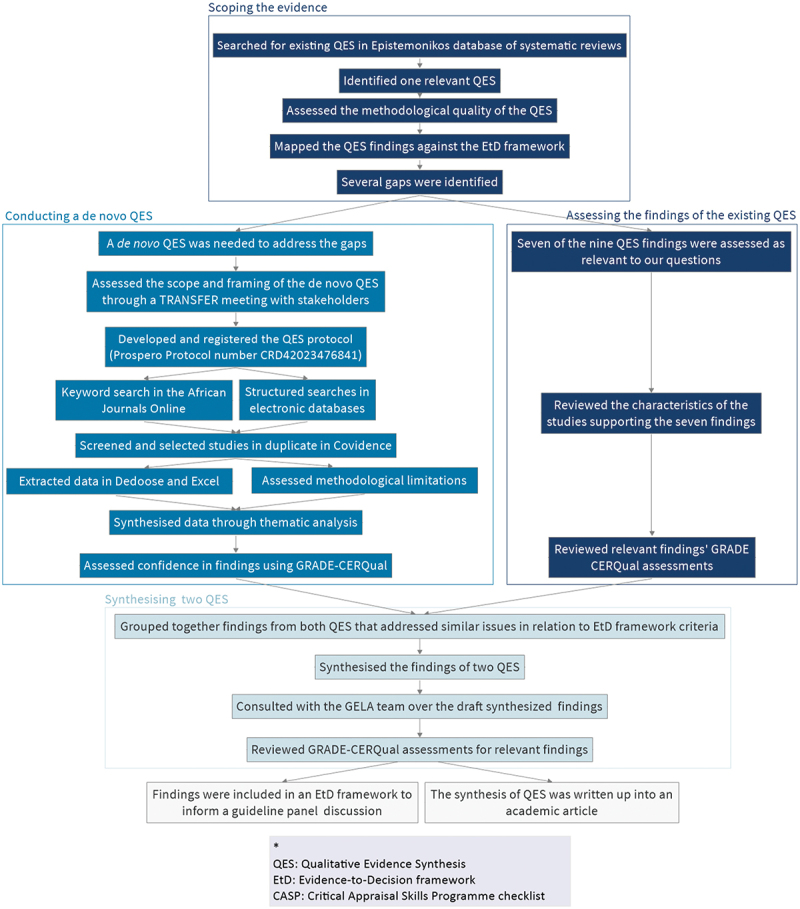
Flowchart representing the method followed in this synthesis of QES*.

An overview of the method is depicted in Figure 1.

### Searching for existing evidence

We searched the Epistemonikos database of systematic reviews [13] for existing QES, broad syntheses of qualitative evidence, mixed-method reviews and structured summaries of qualitative evidence that addressed our research questions; had been published in the last 10 years; and covered studies from any country (see Table 1 and Appendix 1).

**Table 1.** Selection criteria for the scoping search for existing QES [14].

Domains, based on the SPIDER tool for qualitative evidence synthesis (13)	Inclusion	Exclusion
Sample	Healthcare workers working in inpatient wards at secondary and tertiary hospitals, globally	Parents and caregivers Children
Phenomenon of Interest	HCWs’ perceptions of hand hygiene compliance Interventions directed at HCW • audit and monitoring, • communication and reminders, • training and education	Practice of hand hygiene Other infection control-promoting interventions Health system interventions
Design	Any qualitative analysis and synthesis approach used for QES	Quantitative analysis
Evaluation	• Feasibility of implementing HHC interventions • Equity implications of interventions to increase compliance to HH recommendations • Acceptability of HHC interventions to HCWs	Impact on HAIs and other infections Effectiveness Cost
Research type	Qualitative evidence synthesis, broad syntheses of qualitative evidence, mixed-method reviews and structured summaries of qualitative evidence published in the last 10 years	Narrative synthesis Meta-analysis

Results were screened in duplicate and potential disagreements were resolved by consensus. We identified one QES published in 2017 [15] that included 36 reports and provided some evidence on the acceptability, feasibility and equity implications of HHC interventions among HCWs. We assessed its methodology using the EPOC tool [16]. Our mapping of this review’s findings against our research questions showed several important limitations and gaps: the search was run in 2015, the review did not provide findings on the equity impacts of HHC interventions and only included three studies conducted in SSA.

To address these limitations and gaps we carried out a complementary QES (described below). We developed a protocol for this *de novo*-QES according to the Cochrane QES template [17] and registered it in the International Prospective Register of Systematic Reviews, PROSPERO (Protocol number CRD42023476841).

### Review co-production with relevant stakeholders

The scope and framing of this synthesis were informed by a meeting with Nigerian healthcare professionals using the TRANSFER approach [18]. This meeting focused on factors that might affect the transferability of findings from studies conducted in other contexts to the context of interest for use of the QES findings. The TRANSFER meeting focused on the Nigerian context, with questions designed to identify differences and contrasts with other SSA countries. We applied the variations identified between different SSA contexts when assessing the relevance of the QES evidence.

### Criteria for considering studies for the *de novo-QES*

Our *de novo*-QES focused on published qualitative studies of HCWs’ perceptions of selected interventions to improve HHC; that had been carried out in SSA settings; and that had been published since 2015. The inclusion and exclusion criteria are described in Table 2. We decided to exclude mixed-method reviews, as those tend to include thinner qualitative data [19].

**Table 2.** Inclusion/Exclusion criteria for the complementary systematic review of qualitative studies *(de novo QES)*.

	Inclusion	Exclusion
Types of studies	Qualitative primary studies (any design) published since 2015 (date of the Chatfield et al. search).	Quantitative and mixed-method studies. Grey literature (e.g. evaluation reports). Systematic reviews Conference abstracts without a full-text article.
Types of participants	• HCW including clinical staff in direct contact with patients – (e.g. nurses, doctors); management staff having responsibility for hand hygiene and infection prevention (e.g. infection prevention and control focal managers, audit and compliance managers, ward/hospital managers), working in, or in relation to, any wards in secondary and tertiary hospitals in SSA. • Support staff (e.g. cleaning staff, orderlies) working in the same hospital wards.	HCW working in primary care or community facilities. Patients or caregivers.
Types of settings	Any wards in secondary and tertiary hospitals in SSA.	Primary care or community facilities (any country) or hospitals outside of SSA.
Types of interventions	HCW-directed interventions (individual interventions) within the domains of the World Health Organization’s research agenda for hand hygiene: - training and education interventions: (e.g. workshops, lectures, role modelling, focus group discussions, e-learning, other training activities and the provision of information and educational materials);- reminders and communication (e.g. posters, stickers, pictographs or even screen savers which serve as reminders to health workers); - evaluation and feedback interventions (e.g. audits or electronic hand hygiene monitoring systems, surveys and feedback from these systems).	Health system interventions (organisational interventions) such as system change or institutional safety climate. Interventions aiming to encourage patients to remind HCWs about HH. Infrastructure interventions aiming to increase the availability of hand hygiene products and infrastructure.
Phenomenon of interest	HCWs’ perceptions of interventions to improve compliance with HH recommendations.	HCWs’ HH practice or compliance.

### Search methods for identification of primary qualitative studies for the *de novo-QES*

#### Electronic searches

We searched EMBASE, PubMed, and CINAHL from 2015 (see Appendices 2, 3 and 4). Building on the search method implemented by Chatfield et al. [15], we included a combination of subject terms on HH, setting (hospital, health care) and methodology (qualitative). We further added subject terms related to the population (HCW) and geographical area (SSA). We did not apply any language restrictions.

#### Searching other resources

We searched the African Journals Online for articles published since 2015, using the following keywords: ‘hand hygiene’, compliance, Africa, hospitals (see Appendix 5).

We had planned to hand-search the reference list of relevant QES captured by our structured search of electronic databases but no eligible QES were found in our *de novo*-search.

### Study selection for the *de novo-QES*

Two reviewers independently reviewed the title and abstract of the identified studies for eligibility, using the Covidence software platform [20]. We resolved disagreements by consensus and discussion with the wider team. The same two reviewers independently screened the full text of the studies identified as potentially relevant. We recorded the reason for exclusion of studies in Covidence at this stage.

### Sampling strategy for the *de novo-QES*

We had planned to apply a representative sampling strategy to select the studies to include in our synthesis among eligible studies. However, the small number of included studies made this step unnecessary. We included all studies meeting our criteria in the synthesis.

### Data extraction for the *de novo-QES*

We extracted the characteristics of the included studies and recorded these in an Excel spreadsheet, including:

- Descriptive information about the study, including the corresponding author of the study, the title, year of publication, country of study, study setting, population and the aim of the study.
- Descriptive information about the HHC intervention, including type, duration and implementation characteristics when available.

These descriptive data were extracted by one reviewer and checked by a second. Disagreements were resolved by discussion.

Using the qualitative analysis software Dedoose [21] two reviewers further extracted information relevant to the objectives of the synthesis independently. Disagreements were solved by consensus.

### Assessment of the methodological limitations of studies included in the *de novo-QES*

Two reviewers independently assessed the methodological limitations of the included studies using a list of criteria used in several previous reviews [22]. This list was based originally on the Critical Appraisal Skills Programme (CASP)’s tool for qualitative studies [23].

### Data synthesis for the *de novo-QES*

Two reviewers coded the extracted data using a thematic analysis approach in Excel. Codes were then grouped by type of HHC interventions, and then into themes. We synthesised findings from the data under each theme and then matched these with the EtD framework categories of feasibility, acceptability, equity and implementation considerations. Draft findings were shared with all co-authors for review.

### Confidence in the review findings from the *de novo-QES*

e assessed our confidence in the QES findings using the GRADE-CERQual approach and GRADE-CERQual’s online interactive Summary of Qualitative Findings (iSoQ) tool [24]. The review team worked together to apply GRADE-CERQual to each finding, aiming to reach consensus on these assessments.

For the findings shared with the Nigerian guideline panel, our GRADE-CERQual assessments considered, for the assessment of relevance in particular, that the review findings would be used to inform a decision in the Nigerian context [25]. The assessments presented in this paper have been revised to apply to the wider SSA context [26].

### Confidence in the review findings from the Chatfield et al. review

The Chatfield et al. review applied the 2015 version of GRADE-CERQual (which has now been superseded) in ways that were not completely congruent with GRADE-CERQual guidance [27]. It was not possible to re-do the assessments as the paper did not report all the data needed to do so. We reviewed the Chatfield et al. assessments based on the limited information available and acknowledged their application of GRADE-CERQual as a potential limitation. We accounted for this limitation when assessing our confidence in the synthesised findings.

### Synthesising findings from the de novo-QES and the Chatfield review

We extracted from the Chatfield et al. review the findings that related to our synthesis’ aim, together with their GRADE-CERQual assessments. We judged seven of the nine findings to be relevant to our synthesis aim. Given the limited number of findings and contributing studies available, further selection or sampling was judged unnecessary. Then, we juxtaposed the Chatfield and *de novo*-QES findings and brought together in an Excel file the findings that addressed similar issues – for example, findings on HCWs’ views of reminders on HH practices. We also sorted these findings according to the EtD framework criteria that they best addressed (acceptability, feasibility, equity impacts or implementation considerations). We looked across these groups of findings and, where appropriate, synthesised these into an overarching finding. Within each of these findings, we retained references to the original two reviews to ensure traceability of the data.

We shared the draft synthesised findings with the wider GELA project team for feedback and discussion. This led to several changes to the wording of these findings in relation to how best to combine and present these findings within the EtD framework criteria.

To assess confidence in the synthesised findings (see Appendix 13), we considered the GRADE-CERQual assessments for each of the individual review findings that contributed to a synthesised finding. Where the assessments were similar (e.g. all had been assessed as low confidence), we retained that assessment. Where the assessments differed, we looked at the relative contribution of each individual finding to the synthesised finding and made a judgement on an appropriate level of confidence. In doing this, we were conservative and went with the lower level of confidence among the contributing findings unless there were compelling reasons not to do so (e.g. one finding having more weight than the others in the synthesised finding).

In this final GRADE-CERQual assessment we also considered whether the findings from the Chatfield et al. review needed to be downgraded, particularly in relation to relevance to the setting(s) and context of interest.

### Deviation from protocol

Our protocol did not include support staff (e.g. cleaning staff, orderlies) as participants. We decided to include this population during screening as support staff have become part of IPC and HH strategies in the last few years. Some studies did not report data from support staff and health professionals separately.

### Researchers’ reflexivity

Reviewers were invited to reflect on how their background, experience and expectation in the field(s), might have influenced this review when we developed the QES protocol, as described in the Cochrane template for QES protocol [17]. Our review team comprised researchers with diverse backgrounds, including clinical medicine, healthcare research, biochemistry, health systems research and health policy. The level of experience and expertise in QES varied in our team but all authors had worked on or led qualitative evidence syntheses in the past. Because of the diversity present in the team, multiple approaches to qualitative research influenced this work and we needed to build consensus on the one implemented here. To help build this consensus, the authors met regularly to discuss or revise the approach taken for this review and consulted with colleagues from the GELA project.

None of the authors had direct experience with implementing HHC interventions. One author had led HH training in the past but did not work directly with compliance interventions. Two authors also had experience with HH practice in healthcare settings as healthcare professionals. The TRANSFER meeting organised with Nigerian stakeholders in June 2023 was essential for the review team to learn from the experience of HCWs involved in implementing HHC interventions in a SSA country post-COVID.

The authors were unsure of the evidence available on the topic of interest when starting the search for the *de novo* QES. Based on the feedback from the TRANSFER meeting regarding the HH infrastructure and the feasibility of HHC interventions in the Nigerian context, the review team expected to identify several barriers to HHC interventions. The lack of recent studies from the SSA continent was somewhat surprising to the team, especially after the COVID-19 pandemic.

## Results

### De novo QES

#### Description of the studies included in the de novo-QES

**Figure 2.**
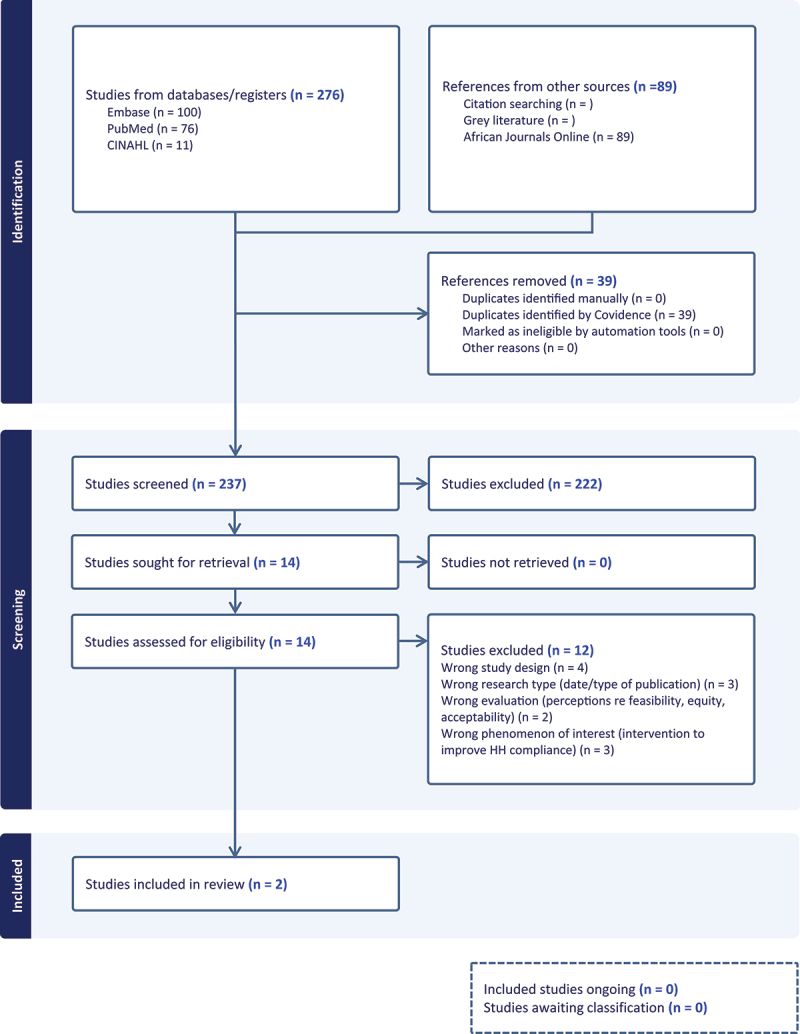
PRISMA flow chart for the search, identification and selection of primary studies in the de novo-QES.

We included two studies (Figure 2), published in 2022 [28] and 2023 [29]. The characteristics of these studies are provided in Appendix 6.

##### Study settings

Both studies were conducted in urban hospitals. One took place in a nursery unit (a referral neonatal ward) in Blantyre, Malawi [29]. The other was conducted in a surgery ward in Cotonou, Benin [28].

##### Participants

Both studies included physicians, nurses and cleaning staff as participants. Additionally, Yehouenou et al. included student nurses, student clinical officers, patient attendants, student nurse/midwife technicians and nurse/midwife technicians [28].

##### Methods

The studies collected data using participant observation [29] and/or interviews [28,29]. Both studies applied a framework analysis of their data.

#### Methodological strengths and limitations of the studies included in the de novo-QES

We assessed that the included studies reported the setting, sampling strategy, participants and ethical considerations adequately, and provided sufficient data to support their findings (see Appendix 8). We had minor concerns about a lack of researcher reflexivity in the articles, although the descriptive nature of these studies mitigated the potential impact and implications of this limitation. The study authors provided general information on their data analysis strategies but did not provide extensive detail on some of their choices or their use of theoretical frameworks.

#### Confidence in the review findings from the de novo-QES

Based on our GRADE-CERQual assessment (see appendix 10), we had moderate confidence in the first five findings, and low confidence in Findings 6 and 7. Our main concerns were the small number of studies (adequacy); the ambiguity of some of the data underlying these findings (for instance, a lack of supporting data in the article, unclear phrasing), affecting the coherence of our findings; and the relevance of the underlying evidence to the diversity of contexts and healthcare systems present across SSA. We also noted that included studies all took place before the COVID-19 pandemic. The pandemic triggered some changes in HHC interventions in our context of interest, thus raising questions as to how relevant and directly applicable these findings are to today’s post-COVID-19 context.

#### Findings from the *de novo-QES*

The findings from our *de novo-*QES are presented in Table 3, with our GRADE-CERQual assessment of confidence. The QES summary of qualitative findings table and evidence profile can be found in Appendix 9 and 10.

**Table 3.** Findings from our *de novo-QES.*

Summarised QES finding	GRADE-CERQual assessment of confidence
**Education and training**	
**Finding 1**: Some HCWs reported being unsure that their HH practice was correct. Healthcare professionals and cleaning staff wanted more training, delivered frequently and that included theoretical and practical aspects of HH. They particularly noted that training in HH technique was lacking. Some nurses also noted that locum nurses did not always get sufficient orientation on infection prevention and control (IPC) (2 studies - [25,26]) ‘*We need a training more and more, moreover and these trainings sessions need to contain more than theoretical information – the HH technic is also important*’ (nurse [25]).	moderate confidence finding
**Finding 2**: HCWs valued training and education for HHC. They acknowledged and knew the role of training in good HH practices and noted the need for more training. Some HCWs also felt that HH training could be an opportunity for discussions on HH best practices (2 studies - [25,26]).	moderate confidence finding
**Finding 3** All staff (including HCWs, and support staff such as cleaners) believed that the full range of staff should receive training on HH to contribute to infection control. Cleaners and hospital attendants were seldom provided information and training opportunities and did not feel empowered to contribute to IPC. One cleaner also noted the importance for IPC of the training and awareness-raising that they had received (2 studies - [25,26]). *‘One of the most important things we’ve done was the training and awareness-raising’ (cleaner* [25]).	moderate confidence finding
**HH reminders for healthcare workers**	
**Finding 4** Most HCWs thought that reminders and posters to support HHC would be useful for HCWs. One nurse noted that these should be placed near all water sources (1 study - [25]). *‘It was necessary to raise awareness and put posters at the water points.’ (nurse*, [25]	moderate confidence finding
**Finding 5** HCWs suggested that posters and other reminders should be implemented together with HH training, and thought that these together could have a considerable effect (1 study - [25])	moderate confidence finding
**Finding 6** HCWs noted the importance of role models to remind staff of HH guidelines and practices and helped improve adherence to guidelines. *‘We would do better with a model, someone who reminds us of the HH practices and guidelines’ (surgeon -* [25]). They also noted that peers in their team could help improve adherence by actively supporting HH practices and reminding colleagues to wash their hands. However, HCWs observed that some staff, including cleaners and hospital attendants, were seldom invited to ward team meetings and so missed important information and training opportunities. This, they suggested, made these staff feel less empowered to contribute to IPC (2 studies - [25,26]).	low confidence finding
**Implementation considerations for hand hygiene interventions**	
**Finding 7** Even when HCWs had a good knowledge of HHC guidelines, heavy workload combined with inadequate HH resources and infrastructure, affected their capacity to implement good HH. HCW described how the lack of or inadequate infrastructure for HH, such as the lack of access to clean water or the poor placement of sinks, hampered good IPC. Poor HHC practice was further exacerbated when workload was heavy (2 study - [25,26]). *‘We are supposed to wash our hands with soap or use a spirit hand rub before and after handling a baby. We are also supposed to clean any cot that a baby has been removed from. (…) But sometimes maybe because of the pressure of work …. it happens that, maybe one baby becomes critically ill and requires urgent attention, we just transfer this baby to another place without considering whether it’s clean or not.’* (nurse midwife in a nursery unit) [26]	low confidence finding

### Chatfield et al. 2017 review

#### Description of the studies included in the Chatfield 2017 review

This review included 36 reports published between 2008 and 2015. The seven findings relevant to our topic of interest were based on 35 reports (see appendix 7).

##### Study settings

The 35 relevant included studies covered all five continents and various levels of healthcare – from clinics to university hospitals. Only three included studies took place in SSA, namely in Uganda [30], Eritrea [31] and Tanzania [32]. These three studies contributed to four Chatfield’s findings. The other studies contributing to the seven findings relevant to our synthesis question came from Europe (eleven studies), North America (ten studies), Asia (five studies), Oceania (three studies), South America (one study) and the Middle East (one study). Three findings did not include any studies from SSA. We highlighted where in the synthesised findings these three Chatfield findings contributed.

As our synthesis question focused on SSA settings, we took differences in settings into consideration when reassessing our confidence in the findings of the Chatfield’s review (see Confidence in the review findings).

##### Participants

The included studies covered various HCWs, but a detailed list of all of these was not provided in the report.

##### Methods

The included studies relied on interviews (individual or in groups), observation or participant narratives during simulations. Chatfield included both mixed-method and qualitative studies. The included approaches as described by the authors of the primary studies included grounded theory, interpretative and descriptive analysis.

#### Methodological strengths and limitations of the studies included in the Chatfield et al. 2017 review

In their assessment of the methodological limitations of included studies, Chatfield et al. [15] assigned a low score to 17 of the 35 reports included in this synthesis due to issues with the reporting of design and study conduct, and a high score to 18 reports assessed as having good quality reporting of design and study conduct.

#### Confidence in the review findings from the Chatfield 2017 review

Among the seven findings from Chatfield et al. [15] relevant to this synthesis, two were assigned high confidence, one moderate and four low confidence. The GRADE-CERQual assessments for these findings are shown in Appendix 11. The main concerns identified in the low confidence finding related to coherence and adequacy, with thinner data and/or fewer studies contributing to this finding. As noted earlier, this QES applied an earlier version of the GRADE-CERQual methodology [27]. Due to concerns over the application of GRADE-CERQual in this QES and also the small number of studies from SSA, we downgraded the relevance of all seven findings and also the overall confidence in the findings (marked in red in Appendices 11 and 12).

#### Findings from the Chatfield QES

Relevant findings from Chatfield et al. are reproduced in Table 4, with our re-assessment of confidence. The summary of qualitative findings table and evidence profile from this review are reproduced in Appendices 11 and 12.

**Table 4.** Relevant findings from Chatfield et al.

Summarised review finding	Number of studies supporting the finding (number of studies from SSA)	Revised GRADE-CERQual assessment of confidence
**H1.** HCW reported HH training is provided on a regular basis, and assert that HCW, as a group, are knowledgeable about HH practice. Some HCW recommended that changes to content or frequency of training might help improve the effectiveness of training, and extension of training to other staff, patients, families and community members might help improve compliance rates.	31 [3]	Moderate
**H2.** HCW reported that their ability to engage in HH is influence by management-related factors that include availability of ample human and hygiene resources along with demonstrated support or priority on hand hygiene originating from upper levels of management. HCW also reported that it is important that HH improvement efforts solicit input from those ‘in the trenches to ensure their support.	29 [3]	Moderate
**M2.** HCW reported that total compliance is not possible or practical given the realities of daily practice. HCW report actual and observed use of gloves as a time-saving alternative although often with less accompanying use of cleaners or sanitisers than is recommended; additionally, HCW reported that gloves are at times changed less frequently than recommended.	23 [1]	Low
**L1.** HCW reported that known surveillance is likely to improve compliance but indicated concerns with accuracy, communication and use of data gathered by electronic monitoring systems. However, there was no consensus among HCW in the sample regarding effectiveness of use of consequences (reward, punishment) in response to HHC.	18 [2]	Very Low
**L2.** HCW reported that compliance with HH is a patient care practice that should be ubiquitous. HCW also reported that over time engaging in hand hygiene becomes an automatic behaviour.	17 (0)	Very Low
**L3.** HCW reported that both HH behaviour and willingness to confront non-compliant others is influenced by hierarchy of staff within facilities. HCW reported that nurses are frequently perceived as beneath physicians; students reported that they perceived themselves as at risk if confronting non-compliant healthcare professionals.	17 (0)	Very Low
**L4.** HCW reported beliefs that are inconsistent with scientific evidence and are not necessarily swayed by presentation of evidence.	16 (0)	Very Low

Table partially reproduced and adapted with permission from Sheryl L. Chatfield, Kristen DeBois, Rachael Nolan, Hannah Crawford, et al., Journal of Infection Prevention [15] pp. 109-112. Copyright © 2016 by (The Authors). Reprinted by Permission of Sage Publications.

### Synthesised findings (de novo-QES and Chatfield QES)

An overview of our confidence assessment for each finding and the contribution of the findings from the de novo and Chatfield et al. QES can be found in Appendix 13.

#### Acceptability of training among healthcare workers

##### Finding 1

While some HCWs noted that HHC becomes an automatic behaviour over time (Chatfield L2), others reported being unsure if their HH practice was correct (*de novo* 1). In studies from African settings, healthcare professionals and cleaning staff acknowledged the role of training in HH practices (*de novo* 2).

*‘Although we receive hand-hygiene training all of the time, we also need to know the practical aspects, not just the theoretical ones’. (physician [29],)*

They wanted more training, delivered frequently, that included locum staff and that covered theoretical and practical aspects of HH, including training in HH techniques (*de novo* 1). Some HCWs also felt that HH training could provide opportunities to discuss HH best practice (*de novo* 2). HCWs from other settings outside SSA, however, suggested that HH practice is natural and should be intrinsically motivated but acknowledged that this was not always the case (Chatfield L2). **[low confidence]**

##### Finding 2

Some studies noted that adequate HH training for HCWs was available but that the content and reach of training could be enhanced (Chatfield H1). Other studies suggested that all cadres should receive training on HH (*de novo* 3). They noted in particular that cleaners and hospital attendants were seldom provided information and training opportunities and did not feel empowered to contribute to IPC (*de novo*3 and Chatfield H1). Some HCWs noted that non-clinical staff were seldom invited to ward team meetings and so missed important information and training opportunities (*de novo* 6).

*‘We are not included in the meetings. I can’t remember when we last had a meeting together’. (patient attendant [29])*

HCWs suggested that training directed at those individuals could potentially help reduce HAI rates (Chatfield H1). **[low confidence]**

#### Acceptability of reminders and communication among healthcare workers

##### Finding 3

Most HCWs thought that reminders and posters to support HH compliance would be useful for HCWs. One nurse noted that these should be placed near all water sources (*de novo* 4). HCWs also suggested that posters and other reminders should be implemented together with HH training, and thought that these together could have a considerable effect (*de novo* 5). **[moderate confidence]**

##### Finding 4

HCWs highlighted the importance of support from higher ranks in the organisation and a supportive institutional environment for HHC, allowing role models and senior staff to remind their colleagues to follow good HH practices.

*‘We can count on the team to improve HHC – they will remind their colleagues to wash their hands’. (surgeon [28])*

However, HCWs also noted that a hierarchical environment may make this type of intervention less effective if more junior or non-clinical staff do not feel empowered to be role models or to remind more senior staff (*de novo* 6 and Chatfield L3). **[low confidence]**

#### Acceptability of monitoring and feedback among healthcare workers

##### Finding 5

The perceived need for and acceptability of monitoring and surveillance interventions for HHC varied across studies. In some studies, HCWs acknowledged that being aware of surveillance, whether via direct monitoring from staff or electronic monitoring (e.g. use of video cameras), might improve HH practice (Chatfield L1). However, in studies conducted outside of SSA, other HCWs questioned whether monitoring was actually needed as these practices should be an instinctive part of care and would become an automatic behaviour with time and experience (Chatfield L2). **[very low confidence]**

##### Finding 6

In studies conducted outside of SSA, HCWs noted that monitoring data should be communicated in a timely manner and in sufficient detail to be useful. They explained that HAI data collected in facilities were not useful when not made available in a timely way or not provided with ample detail. HCWs could lose confidence in data when these lacked contextual information. (Chatfield L4). **[Very low confidence]**

#### Feasibility of HHC interventions

##### Finding 7

Management support, demonstrated by the provision of sufficient human and hygiene resources, was necessary but frequently lacking thus hampering good IPC practice [Chatfield H2, *de novo* 7].

*‘What happens is that we just concentrate on the clinical care of the babies (…). In that way, we can’t consider cleaning as a priority, even changing the water in the suction bottles. We forget to do all that because we have the pressure of work, and we are few nurses on the shift’ (nurse midwife*, [29]).

HCWs noted that asking those involved directly in providing care for their views encouraged ‘buy-in’ from lower levels of the organisational structure [Chatfield H2]. **[low confidence]**

##### Finding 8

Even though HCWs might know what good IPC practices were, workload affected their ability to implement these [Chatfield M2, *de novo* 7]. HCWs noted that they did not have enough time to fully comply with HH policies while managing patient care. Furthermore, some HCWs reported observing that gloves were being used as an alternative to HH practice when facing a heavy workload [Chatfield M2].

*‘We just work without soap. If it’s not there, then there is nothing we can do. We go on working without soap for handwashing or cleaning. But for the gloves, it’s not likely that they run out of stock’ (nurse midwife*, [29]).

Evidence from outside SSA also suggested that HCWs questioned the evidence base linking HH and HAI and the value of recommended HH practices (Chatfield L4). **[low confidence]**

#### Implementation considerations for healthcare facilities and healthcare professionals

We developed a set of questions to help policymakers, hospital staff, managers, and others who are involved in the planning and implementation of HWC-related interventions aimed at improving HH practices for IPC (Table 5). The prompts are informed by our synthesised acceptability and feasibility findings and address important issues linked to the success of the interventions in secondary and tertiary hospital settings in SSA. A lack of detail in the primary studies on the contextual and material factors affecting implementation of HHC interventions prevented us from exploring these issues further in relation to specific contexts.

**Table 5.** Implementation consideration for HCW-related interventions to improve HHC.

**1. How can you ensure that healthcare providers and other staff have access to hand hygiene training?** Do all Healthcare professionals and other relevant staff, such as cleaners, have access to adequate hand hygiene training, delivered on a regular basis? Does this include cadres working in more remote settings? Does available hand hygiene training cover both the theoretical and practical aspects of hand hygiene, including hygiene techniques? Do Healthcare professionals and other relevant staff have opportunities, for instance in trainings and meetings, to discuss hand hygiene best practices? Do Healthcare professionals and other relevant staff, such as cleaners, receive appropriate information (e.g. pamphlets or protocols in plain language) about hand hygiene techniques? Have you considered how to empower cleaners, hospital attendants and other non-clinical staff to contribute to infection protection and control? For example, do these staff think that they can ask for information or remind clinical staff of hand hygiene protocols?
**2. How can you ensure that staff are reminded about the importance of hand hygiene and about hand hygiene technique?** Do Healthcare professionals and other relevant staff (including non-clinical staff) receive or see reminders and other information about hand hygiene, for example at meetings or via posters? Are reminders such as posters placed in appropriate places in the hospital, such as near water sources? Are posters and other reminders made available alongside hand hygiene training? Are managers and other senior staff engaged in supporting and promoting adherence to hand hygiene protocols? Have you considered how to ensure that staff who are seen as role models are engaged in supporting and promoting adherence to recommended hand hygiene protocols and providing information on practices that are not recommended? Have you considered how to ensure that more junior staff and non-clinical staff feel empowered to act as role models and to remind more senior staff of hand hygiene protocols?
**3. How can you use monitoring and feedback to staff to improve adherence to hand hygiene?** Have you discussed with staff whether monitoring and feedback approaches, such as electronic monitoring (e.g. use of video camera), might be a useful and acceptable strategy in your setting? Have you discussed with staff which specific monitoring and feedback approaches might be suitable in your setting? Where monitoring of hand hygiene practices is used, are these monitoring data communicated to all relevant staff in a timely way and with sufficient detail to be useful?
**4. How can you provide appropriate resources and a supportive institutional environment for good hand hygiene** Do staff have access to the resources they need for good infection control practices, including hand hygiene (e.g. running water, soap, alcohol-based sanitizer)? How can you achieve ‘buy-in’ among different levels of staff to implementing appropriate hand hygiene practices? Have managers looked at ways to help ensure that all relevant staff have sufficient time to implement recommended hand hygiene practices?

#### Equity of HHC interventions

We did not find any evidence in either of the reviews synthesised here of factors that impacted directly on equity.

## Discussion

HAI are an important cause of morbidity and mortality in intensive care settings. HH is effective in reducing HAI in NICU settings but can be undermined by poor compliance [4,6]. This QES explored HCW’s perceptions of HHC interventions including training and education; reminders and communication; and audit and feedback. These interventions aim to improve compliance by HCWs with HH recommendations for IPC in hospitalised neonates and infants.

### Key findings

The pre-existing QES by Chatfield et al. identified 35 reports from various healthcare settings on five continents, which informed seven findings relevant to this synthesis. Only three of the studies included by Chatfield et al. took place in SSA. These contributed to four of the seven findings. Our complementary QES identified two more studies conducted in hospitals in SSA.

Our findings highlighted that HH training and education, and reminders and communication interventions were generally acceptable to HCWs but that this population felt that the content, scope and/or target audience of these interventions should be enhanced to improve HHC, to provide better practical HH guidance and be more inclusive of all staff on the ward. Findings on the acceptability of audit and feedback interventions were mixed and may suggest variations across settings. Our synthesis also highlighted key institutional and infrastructure factors that could enhance HHC interventions and their feasibility. These included the importance of an inclusive HHC policy that encompassed all staff; ensuring support from senior staff and leaders, and all-staff buy-in to HHC; and ensuring sufficient human and hygiene resources to implement HH. These factors affecting the feasibility and implementation of HHC interventions were congruent with the barriers and facilitators to HCW’s adherence to broader IPC guidelines highlighted in a recent QES [22].

Our finding on the acceptability of training and reminder interventions is not unexpected, as training and education were given high utility rankings by HCWs, including intensive care staff, in a 2021 study by Lambe et.al [6]. The need to include all cadres of hospital staff in training and education HHC interventions was identified in both QES. A descriptive study [33] from Ghana also found low rates of compliance among non-clinical staff and highlighted their role in mitigating HAI. Our finding highlighting issues around the acceptance of audit and feedback interventions is in line with previous studies in other healthcare settings. For example, a qualitative study in Sweden [34] reported negative perceptions of monitoring at the individual HCW level as these workers were concerned that these data could be used as a form of control by managers. Another study in a UK hospital [35] reported concerns about the accuracy of electronic HH monitoring systems.

Our confidence in these findings ranged from low to very low with only one finding with moderate confidence. This is because of the limited number of studies and the ambiguity of the underlying data. Concerns regarding the relevance of the underlying data to NICUs in the SSA context also contributed to downgrading our confidence in the findings. Low confidence findings may undermine the development of strong policy recommendations to support IPC in hospitals of the region. These limitations highlight the need for more well-conducted qualitative research on HHC interventions in Sub-Saharan Africa and especially in NICU settings. They also point towards a need for a more detailed reporting of findings and their underlying data in both primary qualitative studies and QES on the subject.

Our synthesis reveals several gaps in the research addressing these types of interventions in the SSA region, especially in intensive care settings. Despite known variations in HHC rates, the study of HHC interventions remains limited on the continent. Chatfield et al. included only three studies from SSA and our *de novo*-QES only found two further studies published since 2015, none of which took place in NICU settings. This low number of studies is surprising in the post-COVID-19 era, which has seen HH being further strengthened as part of IPC initiatives. This lack of research may reflect the poor implementation of HHC interventions on the continent. In 2021, WHO found that only 60% of the hospitals surveyed across ten African countries provided training on IPC [36]. Our review also illustrates gaps in the types of HHC interventions studied. We found more evidence on the acceptability and feasibility of training and education interventions than on other types of HHC interventions. This gap makes it difficult to identify HHC interventions responding to the needs and specificities of NICU wards in SSA. We also found little evidence on HCWs’ perception of multifaceted interventions, although these are likely to be more effective than individual HHC interventions [37]. Additionally, the existing studies and QES do not provide sufficient detail on the characteristics and context of HHC interventions to allow us to explore in more detail and compare implementation strategies or contextual factors affecting acceptability and feasibility in SSA. Finally, we did not identify evidence on the potential equity implications of HHC interventions (e.g. unequal access to training opportunities or to HHC promotion resources between staff and facilities).

Despite efforts at promoting HHC, challenges still remain in SSA health settings. These range from insufficient HH supplies of commodities, inadequate HH infrastructure, heavy workloads occasioned by high doctor-to-patient ratios, and lack of training opportunities to the absence of incentives to drive HH practices [38]. As our synthesis demonstrates, these challenges affect the feasibility of HHC interventions. In addition, the lack of IT infrastructure for monitoring and surveillance in many SSA settings adds to the inconsistency in HHC interventions’ implementation and may also explain the thinner data we found regarding monitoring and surveillance interventions. Furthermore, the clinical governance processes aimed at patient safety are yet to be mainstreamed within the organisation of care in health systems in many SSA settings [39]. If such processes are coupled with evidence-based practice informed by clinical guidelines, better patient outcomes may be achieved, assuming that the barriers to HHC interventions identified in this synthesis (e.g. HCC training opportunities for support staff) are addressed.

### Strengths and limitations

#### Novel methodological approaches in the synthesis

This synthesis was developed as part of an evidence-gathering and synthesis process to inform a new national guideline on HCW-related interventions to improve compliance with HH recommendations for IPC in hospitalised neonates and infants in Nigeria [10]. The GRADE-ADOLOPMENT process provided guidance on how to address outdated or incomplete evidence syntheses to inform the acceptability, feasibility, equity or implementation criteria of an evidence-to-decision framework for a guideline recommendation [40]. However, the Chatfield review did not provide sufficient details on the data underlying each finding. The data underlying a QES remain generally unavailable. This makes undertaking an update very challenging. In this synthesis, we made our data and our GRADE-CERQual assessment publicly available on ISoQ [26].

There is little methodological guidance on how to combine or synthesise findings from several QESes or assess their certainty. We adopted an iterative approach that we believe was transparent and credible, and was also suited to the timeline of guideline development. However, more methodological research is needed in this area. Second, uncertainty over how GRADE-CERQual was applied in the Chatfield et al. QES made assessing the confidence of the merged findings more challenging. Fidelity concerns over the application of GRADE-CERQual in QES and its reporting have been identified as known challenges in the uptake of the approach in a recent evaluation [41]. Given the different levels of skills and experience with GRADE-CERQual in our team, the more experienced members of our team provided peer training and guided the discussion around applying this tool to the *de novo*-QES and to the final findings. How to re-assess our certainty in the merged findings was also discussed with the wider GELA project team to build a consensus on the approach. However, in the absence of detailed data extraction from Chatfield et al., a full re-application of GRADE-CERQual approach to both relevant Chatfield findings and our synthesised findings was not feasible, although we initially deemed it preferable.

To help us develop our final findings, we first grouped each QES’ findings according to the key domains of HHC interventions identified by WHO. Within each of these domains, we then examined the findings in relation to the EtD framework criteria to identify common themes. However, thin data, particularly from intensive care settings in SSA, posed a challenge when merging findings from both QESes. We accounted for these difficulties when re-assessing our confidence in our final findings.

#### Ensuring the transferability and relevance of indirect evidence

This synthesis was developed to inform a new guideline on HHC interventions in NICUs in SSA. However, our initial scoping revealed a lack of HHC studies specific to interventions in NICUs, thus motivating our decision not to limit our search to NICUs. We acknowledge there might be variations between different types of wards and within NICUs depending on the setting (e.g. country, hospital level, public/private facilities). However, we took several steps to ensure the relevance and reliability of the evidence we synthesised. The TRANSFER meeting allowed us to identify factors that may affect the transferability of evidence from other wards to a NICU setting. For example, this consultation highlighted the high number of contacts between HCW and NICU patients due to the high number of procedures these patients require, but also some shared concerns and barriers found across different types of wards (e.g. universal importance of HHC in healthcare settings, challenging working conditions and lack of HH infrastructure). We took into account these differences when assessing our confidence in individual findings, particularly when assessing relevance.

## Conclusions

HH is effective in reducing the HAI in NICU setting but can be undermined by poor compliance [4,6]. This QES explored HCW’s perceptions of a range of HHC interventions including training and education; reminders and communication; and audit and feedback. Our synthesis of QES encompasses 37 publications (35 from Chatfield and 2 from our new QES). It highlights that HH training and education, and reminders and communication interventions are acceptable to HCWs. However, they feel that the content, scope and/or target audience of these interventions should be enhanced to improve HHC. For example, HCW expressed the need to expand regular HH training to all categories of staff and provide training that covered both theoretical and practical aspects of HH. Findings on the acceptability of audit and feedback interventions are mixed and may suggest variations across settings, although exploring these variations will require further research and detailed reporting in primary studies. Our synthesis also highlights key institutional and infrastructure factors (such as support at senior level, HHC policy inclusive of all staff) that can enhance HHC interventions and their feasibility. Our confidence in these findings ranges from moderate to very low, owing to the limited number of studies, the ambiguity of the underlying data and concerns over the relevance of the underlying data to the SSA context. This latter concern also highlights the limited number of studies on HHC interventions from SSA, particularly from NICUs where HHC is an essential part of IPC.

Finally, our synthesis piloted a novel methodological approach to synthesise and assess confidence in QES findings in a timely manner to inform national decision-making.

## Supplementary Material

Supplemental Material
