# Supplementary material for: Healthcare workers’ perceptions of health worker-related interventions to improve compliance with hand hygiene recommendations for infection prevention and control in hospitalised neonates and infants in Sub-Saharan Africa: a synthesis of two qualitative evidence syntheses
Source: Glob Health Action. 2026 Feb 17;19(1):2621448. doi: 10.1080/16549716.2026.2621448 (PMC12918283; doi:10.1080/16549716.2026.2621448)
Supplement: Supplemental Material [file ZGHA_A_2621448_SM9129.pdf]

Appendix 1: Search for systematic reviews of qualitative evidence in Epistemonikos.

Date of search: 21st July 2023

1	Hand Hygiene	(title:((title:("hand-hygiene" OR "hand hygiene") OR abstract:("hand-hygiene" OR "hand hygiene")) AND (title:(compliance OR adherence) OR abstract:(compliance OR adherence))) OR abstract:((title:("hand-hygiene" OR "hand hygiene") OR abstract:("hand-hygiene" OR "hand hygiene"))
2	Compliance / Adherence	(title:(compliance OR adherence) OR abstract:(compliance OR adherence))))
3	Qualitative and Mixed method	interview* OR qualitative OR "focus group" OR mixed OR "mixed method" OR MMR OR themat* OR ethnograph*
Filter	Last 10 years [filter]	
Filter	Type of study: Systematic review, Synthesis, Summary [filter]	

1 AND 2 AND 3

(title:((title:((title:("hand-hygiene" OR "hand hygiene") OR abstract:("hand-hygiene" OR "hand hygiene")) AND (title:(compliance OR adherence) OR abstract:(compliance OR adherence))) OR abstract:((title:("hand-hygiene" OR "hand hygiene") OR abstract:("hand-hygiene" OR "hand hygiene")) AND (title:(compliance OR adherence) OR abstract:(compliance OR adherence))))) OR abstract:((title:((title:("hand-hygiene" OR "hand hygiene") OR abstract:("hand-hygiene" OR "hand hygiene")) AND (title:(compliance OR adherence) OR abstract:(compliance OR adherence))) OR abstract:((title:("hand-hygiene" OR "hand hygiene") OR abstract:("hand-hygiene" OR "hand hygiene")) AND (title:(compliance OR adherence) OR abstract:(compliance OR adherence)))))) AND (title:(interview* OR qualitative OR "focus group" OR mixed OR "mixed method" OR MMR OR themat* OR ethnograph*) OR abstract:(interview* OR qualitative OR "focus group" OR mixed OR "mixed method" OR MMR OR themat* OR ethnograph*))

Total: 109
Limited to

- last 10 years: 90
- last 10 years AND systematic review: 14
- last 10 years AND broad synthesis: 1
- last 10 years AND structured summary: 0

Appendix 2: Search strategy for the de novo-QES - EMBASE

Date of search: 03/10/2023

('hand washing'/exp OR 'hand washing' OR 'disinfectant agent'/exp OR 'disinfectant agent' OR 'hand hygiene':ab,ti OR 'disinfect*or rub':ab,ti OR wash*:ab,ti OR sanitis*:ab,ti OR handwashing*:ab,ti OR sanitiz*:ab,ti OR gel:ab,ti) AND ('hospital' OR 'hospital'/exp OR hospital OR hospital:ab,ti OR 'health care facility':ab,ti OR 'health care':ab,ti OR ward*:ab,ti OR 'intensive care':ab,ti OR picu:ab,ti OR 'neonatal intensive care unit':ab,ti OR nicu:ab,ti OR 'pediatric intensive care unit':ab,ti) AND (('africa south of the sahara'/exp OR 'africa south of the sahara' OR ((2aniti*:ab,ti OR angola:ab,ti OR benin:ab,ti OR 2anitize:ab,ti OR 'burkina faso':ab,ti OR 2anitiz:ab,ti OR 2anitize:ab,ti OR 'canary islands':ab,ti OR 'cape verde':ab,ti OR 'central africa':ab,ti OR chad:ab,ti OR comoros:ab,ti OR congo:ab,ti OR 'democratic republic congo':ab,ti OR 2anitize:ab,ti OR 'equatorial guinea':ab,ti OR 2anitiz:ab,ti OR 2anitize:ab,ti OR gabon:ab,ti OR gambia:ab,ti OR ghana:ab,ti OR guinea:ab,ti OR 'guinea bissau':ab,ti OR 'cote d`ivoire':ab,ti OR 'ivory coast':ab,ti OR kenya:ab,ti OR 2anitiz:ab,ti OR 2anitiz:ab,ti OR 2anitize22:ab,ti OR 2aniti:ab,ti OR mali:ab,ti OR 2anitize22:ab,ti OR 2anitize2:ab,ti OR 2anitize22:ab,ti OR mocambique:ab,ti OR 2anitiz:ab,ti OR niger:ab,ti OR 2anitiz:ab,ti OR principe:ab,ti OR 2aniti:ab,ti OR 'sao tome':ab,ti) AND principe:ab,ti) OR 2anitiz:ab,ti OR 2anitize22:ab,ti OR 'sierra leone':ab,ti OR 2anitiz:ab,ti OR 'south africa':ab,ti OR 'saint helena':ab,ti OR sudan:ab,ti OR eswatini:ab,ti OR 2anitize2:ab,ti OR 2anitize:ab,ti OR togo:ab,ti OR 2aniti:ab,ti OR 'western sahara':ab,ti OR zaire:ab,ti OR 2aniti:ab,ti OR 2anitize:ab,ti OR 'central africa':ab,ti OR 'central african':ab,ti OR 'west africa':ab,ti OR 'west african':ab,ti OR 'western africa':ab,ti OR 'western african':ab,ti OR 'east africa':ab,ti OR 'east african':ab,ti OR 'eastern africa':ab,ti OR 'eastern african':ab,ti OR 'north africa':ab,ti OR 'north african':ab,ti OR 'south african':ab,ti OR 'southern africa':ab,ti OR 'southern african':ab,ti OR 'sub 2anitiz africa':ab,ti OR 'sub 2anitiz african':ab,ti OR 'subsaharan africa':ab,ti OR 'subsaharan african':ab,ti) NOT ('guinea pig':ab,ti OR 'guinea pigs':ab,ti OR 'aspergillus niger':ab,ti)) AND ('health care personnel'/exp OR 'health care personnel' OR 'physician'/exp OR physician OR 'nurse'/exp OR nurse OR 'clinician'/exp OR clinician OR 'medical staff'/exp OR 'medical staff' OR 'paramedical personnel'/exp OR 'paramedical personnel' OR 'healthcare professional*':ab,ti OR 'healthcare worker*':ab,ti OR 'health personnel*':ab,ti OR 'healthcare provider*':ab,ti OR doctor*:ab,ti OR nurse*:ab,ti OR clinician*:ab,ti OR 'healthcare assistant*':ab,ti OR 'medical staff':ab,ti OR ((health?care NEAR/2 (staff OR assistant* OR personnel OR provider* OR professional*)):ab,ti)) AND ('qualitative research'/exp OR 'qualitative research' OR 'interview' OR 'interview'/exp OR interview OR 'focus group'/exp OR 'focus group') AND [2015-2023]/py	100

Appendix 3: Search strategy for the de novo-QES - PubMed

Date of search: 03/10/2023

(“Hand Hygiene”[Title/Abstract] OR “disinfect*”[Title/Abstract] OR “rub”[Title/Abstract] OR “wash*”[Title/Abstract] OR “handwashing”[Title/Abstract] OR “sanitize*”[Title/Abstract] OR “sanitise*”[Title/Abstract] OR “gel”[Title/Abstract] OR “Hand Hygiene”[MeSH Terms] OR “Hand Disinfection”[MeSH Terms]) AND (“hospital*”[Title/Abstract] OR “Health facility”[Title/Abstract] OR “Health facilities”[Title/Abstract] OR “health care setting*”[Title/Abstract] OR “healthcare setting*”[Title/Abstract] OR “health care 3anitize*”[Title/Abstract] OR “ward*”[Title/Abstract] OR “intensive care unit*”[Title/Abstract] OR “PICU”[Title/Abstract] OR “ICU”[Title/Abstract] OR “NICU”[Title/Abstract] OR “Hospitals”[MeSH Terms] OR “Health facilities”[MeSH Terms:noexp]) AND (((“3aniti*”[Text Word] OR “Angola”[Text Word] OR “Benin”[Text Word] OR “Botswana”[Text Word] OR “Burkina Faso”[Text Word] OR “Burundi”[Text Word] OR “Cameroon”[Text Word] OR “Canary Islands”[Text Word] OR “Cape Verde”[Text Word] OR “Central African Republic”[Text Word] OR “Chad”[Text Word] OR “Comoros”[Text Word] OR “Congo”[Text Word] OR “Democratic Republic of Congo”[Text Word] OR “Djibouti”[Text Word] OR “Equatorial Guinea”[Text Word] OR “Eritrea”[Text Word] OR “Ethiopia”[Text Word] OR “Gabon”[Text Word] OR “Gambia”[Text Word] OR “Ghana”[Text Word] OR “Guinea”[Text Word] OR “Guinea Bissau”[Text Word] OR “Ivory Coast”[Text Word] OR “Cote d’Ivoire”[Text Word] OR “Kenya”[Text Word] OR “Lesotho”[Text Word] OR “Liberia”[Text Word] OR “Madagascar”[Text Word] OR “Malawi”[Text Word] OR “Mali”[Text Word] OR “Mauritania”[Text Word] OR “Mauritius”[Text Word] OR “Mozambique”[Text Word] OR “Mocambique”[Text Word] OR “Namibia”[Text Word] OR “Niger”[Text Word] OR “Nigeria”[Text Word] OR “Principe”[Text Word] OR “Rwanda”[Text Word] OR “Sao Tome”[Text Word] OR “Senegal”[Text Word] OR “Seychelles”[Text Word] OR “Sierra Leone”[Text Word] OR “Somalia”[Text Word] OR “South Africa”[Text Word] OR “St Helena”[Text Word] OR “Sudan”[Text Word] OR “Swaziland”[Text Word] OR “Eswatini”[Text Word] OR “Tanzania”[Text Word] OR “Togo”[Text Word] OR “Uganda”[Text Word] OR “Western Sahara”[Text Word] OR “Zaire”[Text Word] OR “Zambia”[Text Word] OR “Zimbabwe”[Text Word] OR “Central Africa”[Text Word] OR “Central African”[Text Word] OR “West Africa”[Text Word] OR “West African”[Text Word] OR “Western Africa”[Text Word] OR “Western African”[Text Word] OR “East Africa”[Text Word] OR “East African”[Text Word] OR “Eastern Africa”[Text Word] OR “Eastern African”[Text Word] OR “North Africa”[Text Word] OR “North African”[Text Word] OR “South African”[Text Word] OR “Southern Africa”[Text Word] OR “Southern African”[Text Word] OR “sub Saharan Africa”[Text Word] OR “sub Saharan African”[Text Word] OR “subSaharan Africa”[Text Word] OR “subSaharan African”[Text Word]) NOT (“guinea pig”[Text Word] OR “guinea pigs”[Text Word] OR “aspergillus niger”[Text Word])) OR “Africa South of the Sahara”[MeSH Terms]) AND (“healthcare professional*”[Title/Abstract] OR “health care professional*”[Title/Abstract] OR “healthcare worker*”[Title/Abstract] OR “health care worker*”[Title/Abstract] OR “health worker*”[Title/Abstract] OR “Health Personnel”[Title/Abstract] OR “Health Care Personnel”[Title/Abstract] OR “Healthcare Personnel”[Title/Abstract] OR “health care provider*”[Title/Abstract] OR “healthcare provider*”[Title/Abstract] OR “health professional*”[Title/Abstract] OR “doctor*”[Title/Abstract] OR “physician*”[Title/Abstract] OR “nurse*”[Title/Abstract] OR “clinician*”[Title/Abstract] OR “healthcare assistant*”[Title/Abstract] OR “health care assistant*”[Title/Abstract] OR “medical staff”[Title/Abstract] OR “Health Personnel”[MeSH Terms] OR “personnel, hospital”[MeSH Terms]) AND (“qualitative”[Title/Abstract] OR “focus group*”[Title/Abstract] OR “interview*”[Title/Abstract] OR “Qualitative Research”[MeSH Terms] OR “Focus Groups”[MeSH Terms] OR “Interviews as Topic”[MeSH Terms]) AND 2015/09/01:2023/09/29[Date – Publication]	76

Appendix 4: Search strategy for the de novo-QES - CINAHL

Date of search: 03/10/2023

#	Query	Limiters/Expanders	Last Run Via	Results
S6	S1 AND S2 AND S3 AND S4 AND S5	Expanders – Apply equivalent subjects<br>Search modes – Boolean/Phrase	Interface – EBSCOhost Research Databases<br>Search Screen – Advanced Search<br>Database – CINAHL Complete	11
S5	MW (qualitative research or qualitative study or qualitative methods or interview or focus group) OR AB (qualitative research or qualitative study or qualitative methods or interview or focus group)	Expanders – Apply equivalent subjects<br>Search modes – Boolean/Phrase	Interface – EBSCOhost Research Databases<br>Search Screen – Advanced Search<br>Database – CINAHL Complete	326,319
S4	MW (healthcare workers or nurses or medical workers or healthcare professionals) OR AB (healthcare workers or nurses or medical workers or healthcare professionals) OR AB ("Health Personnel" OR "Health Care Personnel" OR "Healthcare Personnel" OR "health care provider*" OR "healthcare provider*" OR "health professional*" OR "doctor*" OR "physician*" OR "nurse*" OR "clinician*" OR "healthcare assistant*" OR "health care assistant*" OR "medical staff" OR "Health Personnel" OR "hospital personnel"))	Expanders – Apply equivalent subjects<br>Search modes – Boolean/Phrase	Interface – EBSCOhost Research Databases<br>Search Screen – Advanced Search<br>Database – CINAHL Complete	794,746
S3	MW (sub sanitiz saniti or sub-saharan 5aniti or sub sahara or sub-sahara) OR AB (Africa* OR Angola OR Benin OR Botswana OR "Burkina Faso" OR Burundi OR Cameroon OR "Canary Islands" OR "Cape Verde" OR "Central African Republic" OR Chad OR Comoros OR Congo OR "Democratic Republic of Congo" OR Djibouti OR "Equatorial Guinea" OR Eritrea OR Ethiopia OR Gabon OR Gambia OR Ghana OR Guinea OR "Guinea Bissau" OR "Ivory Coast" OR "Cote d'Ivoire" OR Kenya OR Lesotho OR Liberia OR Madagascar OR Malawi OR Mali OR Mauritania OR Mauritius OR Mozambique OR Mocambique OR Namibia OR Niger OR Nigeria OR Principe OR Rwanda OR "Sao Tome" OR Senegal OR Seychelles OR "Sierra Leone" OR Somalia OR "South Africa" OR "St Helena" OR Sudan OR Swaziland OR Eswatini OR Tanzania OR Togo OR Uganda OR "Western Sahara" OR Zaire OR Zambia OR Zimbabwe OR "Central Africa" OR "Central African" OR "West Africa" OR "West African" OR "Western Africa" OR "Western African" OR "East Africa" OR "East African" OR "Eastern Africa" OR "Eastern African" OR "North Africa" OR "North African" OR "South African" OR "Southern Africa" OR "Southern African" OR "sub Saharan Africa" OR "sub Saharan African" OR "subSaharan Africa" OR "subSaharan African") NOT AB ("guinea pig" OR "guinea pigs" OR "aspergillus niger")	Expanders – Apply equivalent subjects<br>Search modes – Boolean/Phrase	Interface – EBSCOhost Research Databases<br>Search Screen – Advanced Search<br>Database – CINAHL Complete	106,453

S2	MW (hospital or acute setting or inpatient or ward) OR AB (hospital or acute setting or inpatient or ward) OR AB ("Health facility" OR "Health facilities" OR "health care setting*" OR "healthcare setting*" OR "health care 6anitize*" OR "intensive care unit*" OR "PICU" OR "ICU" OR "NICU" OR "Hospitals"OR "Health facilities")	Expanders – Apply equivalent subjects<br>Search modes – Boolean/Phrase	Interface – EBSCOhost Research Databases<br>Search Screen – Advanced Search<br>Database – CINAHL Complete	573,114
S1	MW (hand hygiene or handwashing or hand washing or hand disinfection) OR AB (hand hygiene or handwashing or hand washing or hand disinfection) OR AB ("wash" or disinfect* OR gel or 6anitize* or sanitis*)	Expanders – Apply equivalent subjects<br>Search modes – Boolean/Phrase	Interface – EBSCOhost Research Databases<br>Search Screen – Advanced Search<br>Database – CINAHL Complete	30,539

Appendix 5: Search strategy for the de novo-QES - African Journals online

Date of the search: 18 oct. 2023

Keywords: “hand hygiene” AND compliance AND Africa AND hospitals

Search results: 185

Search results since 2015: 89

Appendix 6. Characteristics of included studies – de novo-QES

Study#	Reference	Study title	Study aim	Country, classification by income level	Participants	Setting	Type of hand hygiene intervention	Description of the intervention (key features of the intervention, single/combined intervention)	Implementation characteristics of the intervention (Important factors or conditions of implementation of the intervention studied).	Duration of HHC intervention
1	Mangochi et al. 2023	A qualitative study exploring hand hygiene practices in a neonatal unit in Blantyre, Malawi: implications for controlling healthcare-associated infections	To identify barriers to optimal Infection Prevention and Control, focusing on hand hygiene.	Malawi, low-income country	medical doctors, clinical officers, student clinical officers, cleaners, patient attendants, student nurse/midwife technicians, nurse/midwife technicians and state registered nurse.	Chatinkha nursery unit (referral neonatal unit), Queen Elizabeth Central Hospital (QECH) in Blantyre	Training and Education	Single	Not reported	Not reported
2	Yehouenou et al. 2022	Understanding Hand Hygiene Behaviour in a Public Hospital in Benin Using the Theoretical Domain Frameworks: The First Step for Designing Appropriate Interventions	To identify several behavioural constructs aligned with the Theoretical Domains Framework that can be targeted and used for the development of new hand-hygiene interventions.	Benin, Lower-middle income country	Physicians, nurses, surgeons, cleaning staff.	Surgery ward in a confessional hospital in Cotonou	Training and education; Posters and reminders	Combined	Not reported	Not reported

Appendix 7. Characteristics of included studies – Chatfield review

Please note that this table includes only the studies that contributed to the findings relevant to this synthesis.

Source	Location	Participants	Data collection	Facility Type
Barrett and Randle (2008)	UK	Nursing students	Individual interviews	Community or other non-teaching hospital
Boscart et al. (2012)	Canada	Multiple healthcare workers	Individual interviews	Community or other non-teaching hospital
Boog et al. (2013)	Europe	Multiple HCW	Observation and interviews	Community or other non-teaching hospital
Brown et al. (2008)	UK	Nurses	Individual interviews	Community or other non-teaching hospital
Bryce et al. (2014)	Canada	Regional directors of infection control	Individual interviews	Community or other non-teaching hospital
Cole (2009)	UK	Nursing students	Observation and interviews	Community or other non-teaching hospital
Dawson (2015)	UK	Nurses	Observation and interviews	Community or other non-teaching hospital
De Abreu Botene and Pedro (2014)	South America	Multiple healthcare workers	Observation and interviews	Community or other non-teaching hospital
Dixit et al. (2012)	Canada	Physicians	Individual interviews	Community or other non-teaching hospital
Dyson et al. (2011)	UK	Multiple healthcare workers	Observation and interviews	Community or other non-teaching hospital
Ellingson et al. (2011)	USA	Multiple healthcare workers	Group interviews	Mix of teaching and non-teaching hospitals
Erasmus et al. (2009)	Europe	Multiple healthcare workers	Individual and group interviews	Mix of teaching and non-teaching hospitals
Eveillard et al. (2013)	Europe	Multiple healthcare workers	Observation and interviews	Community or other non-teaching hospital
Hill et al. (2013)	USA	Multiple healthcare workers	Group interviews	Mix of hospitals and clinics
Jang et al. (2010a)	Canada	Physicians	Group interviews	Community or other non-teaching hospital
Jang et al. (2010b)	Canada	Multiple healthcare workers	Group interviews	Community or other non-teaching hospital
Johnson et al. (2012)	Central America	Multiple healthcare workers	Group interviews	Community or other non-teaching hospital
Jones et al. (2015)	Africa - Tanzania	Multiple healthcare workers	Group interviews	Other (mix of facility and community-based carers)
Joshi et al. (2012)	Asia	Multiple healthcare workers	Group interviews	Community or other non-teaching hospital
Lee (2013)	UK	Nursing students	Participant narrative during simulation	Other (academic setting)
Lusardi (2007)	UK	Nursing students	Individual interviews	Community or other non-teaching hospital
Marjadi and McLaws (2010)	Asia	Multiple healthcare workers	Observation and interviews	Mix of hospitals and clinics
McInnes (2014)	Australia	Administrators and clinical supervisors	Individual interviews	Community or other non-teaching hospital
McLaws et al. (2012)	Middle East	Multiple healthcare workers	Individual and group interviews	Mix of teaching and non-teaching hospitals
Nichols and Badger (2008)	UK	Multiple healthcare workers	Observation and interviews	Community or other non-teaching hospital
Nicol et al. (2009)	Australia	Multiple healthcare workers	Observation and interviews	Community or other non-teaching hospital
Pan et al. (2014)	Asia	Medical students	Observation and interviews	Community or other non-teaching hospital
Patterson et al. (2014)	USA	Multiple HCW	Individual and group interviews	Community or other non-teaching hospital
Salmon & McLaws (2015)	Asia	Multiple healthcare workers	Group interviews	Community or other non-teaching hospital
Samuel et al. (2005)	Africa - Eritrea	Multiple healthcare workers	Observation and interviews	Community or other non-teaching hospital

Seibert et al. (2014)	USA	Multiple healthcare workers	Individual interviews	Community or other non-teaching hospital
Wasswa et al. (2015)	Africa - Uganda	Multiple healthcare workers	Observation and interviews	Mix of hospitals and clinics
White et al. (2015)	Australia	Nurses	Group interviews	Community or other non-teaching hospital
Yuan et al. (2009)	Asia	Multiple healthcare workers	Individual interviews	Community or other non-teaching hospital
Zimmerman et al. (2013)	Canada	Multiple healthcare workers	Individual interviews	Community or other non-teaching hospital

Table extracted from: Chatfield, S. L., DeBois, K., Nolan, R., Crawford, H., & Hallam, J. S. (2017). Hand hygiene among healthcare workers: A qualitative meta summary using the GRADE-CERQual process. *Journal of infection prevention*, *18*(3), 104-120.

Table partially reproduced and adapted with permission from Sheryl L. Chatfield, Kristen DeBois, Rachael Nolan, Hannah Crawford, et al., Journal of Infection Prevention [15] pp. 109-112. Copyright © 2016 by (The Authors). Reprinted by Permission of Sage Publications.

Appendix 8 Methodological strengths and limitations of included studies for the de novo-QES.

**Study ID**	**Was the setting(s) and context adequately described**	**Was the sampling strategy appropriate and described?**	**Was the data collection strategy appropriate and described?**	**Was the data analysis appropriate and described?**	**Were the findings (claims made) supported by evidence?**	**Is there evidence of researcher reflexivity?**	**Have ethical issues been taken into consideration?**	**Any other concerns**	**Overall assessment of methodological limitations**
Mangochi 2023	Yes	Yes	Yes	Yes	No	Unclear	Yes	Some elements of the methodology lack details.	Moderate
Yehouenou 2022	Yes	Yes	Yes	Yes	No	No	Yes	Themes in Table 2 are unclear not all supported by data.	Moderate

Appendix 9: Summary of Qualitative Findings table for the de novo-QES

#	Summarised review finding	GRADE-CERQual Assessment of confidence	Explanation of GRADE-CERQual Assessment	References
1	Finding 1: Some HCWs reported being unsure that their HH practice was correct. Healthcare professionals and cleaning staff wanted more training, delivered frequently and that included theoretical and practical aspects of HH. They particularly noted that training in HH technique was lacking. Some nurses also noted that locum nurses did not always get sufficient orientation on IPC (2 studies)	Moderate confidence	Minor concerns regarding methodological limitations, Moderate concerns regarding coherence, Moderate concerns regarding adequacy, and Minor concerns regarding relevance. There is uncertainty due to the limited number of studies and the ambiguity of some of the data or imprecision in the information available. Due to the low number of studies included, we cannot be certain that this finding would be applicable to all NICU settings in Sub-Saharan Africa, although feedback from practitioners tells us that variations across countries for this level and type of care might be limited. We do note that this finding is based on studies carried out pre-COVID, which raises uncertainty as to how applicable it is today.	Mangochi et al. 2023; Yehouenou et al. 2022;
2	Finding 2: HCWs valued training and education for HH compliance. They acknowledged and knew the role of training in good HH practices and noted the need for more training. Some HCWs also felt that HH training could be an opportunity for discussions on HH best practices. (2 studies)	Moderate confidence	Minor concerns regarding methodological limitations, No/Very minor concerns regarding coherence, Moderate concerns regarding adequacy, and Minor concerns regarding relevance We are concerned by the adequacy component and the lack of post-COVID, multi-setting data that would make us confident in the applicability of this finding across facilities and countries in Sub-Saharan Africa in today's world.	Mangochi et al. 2023; Yehouenou et al. 2022;
3	Finding 3 – training for all staff: All staff (including HCWs, and support staff such as cleaners) believed that all range of staff should receive training on HH to contribute to infection control. Cleaners and hospital attendants were seldom provided information and	Moderate confidence	Minor concerns regarding methodological limitations, No/Very minor concerns regarding coherence, Moderate concerns regarding adequacy, and Minor concerns regarding relevance The lack of studies post COVID and how the situation has evolved since then raises	Mangochi et al. 2023; Yehouenou et al. 2022;

#	Summarised review finding	GRADE-CERQual Assessment of confidence	Explanation of GRADE-CERQual Assessment	References
	training opportunities and did not feel empowered to contribute to IPC. One cleaner also noted the importance for IPC of the training and awareness-raising that they had received.(2 studies)		questions as to how applicable the finding still is applicable to NICUs today. We would expect that the stronger focus on HH further to COVID may have affected the opportunities for training for all staff across the continent.	
4	Finding 4 – reminders and posters: Most HCWs thought that reminders and posters to support HH compliance would be useful for HCWs. One nurse noted that these should be placed near all water sources. (1 study).	Moderate confidence	Minor concerns regarding methodological limitations, No/Very minor concerns regarding coherence, Minor concerns regarding adequacy, and Minor concerns regarding relevance Even though we don't have any major concerns, the data is very limited (1 study) which affect how confident we can be in the finding in general.	Yehouenou et al. 2022;
5	Finding 5 –combining and incorporating posters into training: HCWs suggested that posters and other reminders should be implemented together with HH training, and thought that these together could have a considerable effect. (1 study)	Moderate confidence	No/Very minor concerns regarding methodological limitations, Minor concerns regarding coherence, Moderate concerns regarding adequacy, and No/Very minor concerns regarding relevance. The data supporting this finding is very limited (1 study, 3 excerpts). However, we do not expect significant variations or differences across Sub-Saharan African countries that would warrant further downgrading this finding.	Yehouenou et al. 2022;
6	Finding 6 – role models and peer influence - to build a supportive institutional and management environment: HCWs noted the importance of role models to remind staff of hand hygiene guidelines and practices and helped improve adherence to guidelines. They also noted that peers in their team could help improve adherence by actively supporting hand hygiene practices and reminding colleagues to wash their hands. However, HCWs observed that some staff, including cleaners and	Low confidence	No/Very minor concerns regarding methodological limitations, No/Very minor concerns regarding coherence, Moderate concerns regarding adequacy, and Minor concerns regarding relevance Limited number of studies, thin data in each study and also questions about whether part of the finding is directly applicable to all the Sub-Saharan African contexts today (post-pandemic)	Mangochi et al. 2023; Yehouenou et al. 2022;

#	Summarised review finding	GRADE-CERQual Assessment of confidence	Explanation of GRADE-CERQual Assessment	References
	hospital attendants, were seldom invited to ward team meetings and so missed important information and training opportunities. This, they suggested, made these staff feel less empowered to contribute to IPC. (2 studies)			
7	Finding 7: Even when HCWs had a good knowledge of HHC guidelines, heavy workload combined with inadequate HH resources and infrastructure affected their capacity to implement good HH. HCW described how the lack of or inadequate infrastructure for HH, such as the lack of access to clean water or the poor placement of sinks, hampered good infection prevention and control. Poor HHC practice was further exacerbated when workload was heavy. (2 studies)	Low confidence	Minor concerns regarding methodological limitations, No/Very minor concerns regarding coherence, Moderate concerns regarding adequacy, and Minor concerns regarding relevance. This is because of the small number of studies from only two countries on the continent. However, this is a descriptive finding and we anticipate that many African countries have hospitals that experience similar constraints as those highlighted in these 2 studies.	Mangochi et al. 2023; Yehouenou et al. 2022;

Appendix 10: Qualitative Evidence Profile for the de novo-QES

#	Summarised review finding	Methodological limitations	Coherence	Adequacy	Relevance	GRADE-CERQual assessment of confidence	References
1	Finding 1: Some HCWs reported being unsure that their HH practice was correct. Healthcare professionals and cleaning staff wanted more training, delivered frequently and that included theoretical and practical aspects of HH. They particularly noted that training in HH technique was lacking. Some nurses also noted that locum nurses did not always get sufficient orientation on IPC (2 studies)	Minor concerns<br><br>**Explanation:** Minor concerns regarding methodological limitations because both studies lack information on reflexivity of the researcher and have somewhat unclear reporting on either the methodology or the use of a theoretical framework. However, we do not see these limitation deeply	Moderate concerns<br><br>**Explanation:** Moderate concerns regarding coherence because of issues around the ambiguity of the data. We were sometimes unsure of what the studies meant in some of the excerpts (f. ex: what "practical/technical training" referred to) We were sometime unsure of who made the statements supporting the findings (type of	Moderate concerns<br><br>**Explanation:** Moderate concerns regarding adequacy because we only have two studies with fairly thin data from only two African countries. This creates some uncertainty as to whether this finding would be applicable to all NICU settings in Sub-Saharan Africa and there may also be differences	Minor concerns<br><br>**Explanation:** Minor concerns regarding partial relevance because: - There may have been changes further to COVID (as this event has led to more intensive training). - The healthcare settings: 1 of the studies matches the context of the review (NICU) , the other doesn't (but is in a surgery wards where HH is more likely to be	Moderate confidence<br><br>**Explanation:** Minor concerns regarding methodological limitations, Moderate concerns regarding coherence, Moderate concerns regarding adequacy, and Minor concerns regarding relevance. There is uncertainty due to the limited number of studies and the ambiguity of some of the data or imprecision in the information available. Due to the low number of studies included, we cannot be certain that this finding would be applicable to all NICU settings in Sub-Saharan Africa, although feedback from practitioners tells us that variations across countries for this level and type of care might be limited. We do note that this finding is based on	Mangochi et al. 2023; Yehouenou et al. 2022;

#	Summarised review finding	Methodological limitations	Coherence	Adequacy	Relevance	GRADE-CERQual assessment of confidence	References
		affecting this finding.	stakeholders) We have no concerns over some of the data contradicting the finding.	between the public and the private sectors.	strictly applied). In addition, the studies are from two poorer African countries - Institutional arrangements: there might be differences in how formalised procedures are - Use of locum/temporary staff: the use of locum staff may vary across countries and across the private and public sectors. The situation highlighted by Mangochi would not be applicable to all settings - We are unsure how training is	studies carried out pre-COVID, which raises uncertainty as to how applicable it is today.	

#	Summarised review finding	Methodological limitations	Coherence	Adequacy	Relevance	GRADE-CERQual assessment of confidence	References
					implemented across different settings and the extent to which the training experience described here is generalisable.		
2	Finding 2: HCWs valued training and education for HH compliance. They acknowledged and knew the role of training in good HH practices and noted the need for more training. Some HCWs also felt that HH training could be an opportunity for discussions on HH best practices. (2 studies)	Minor concerns<br><br>**Explanation:** Minor concerns regarding methodological limitations because both studies lack information on reflexivity of the researcher and have somewhat unclear reporting on either the methodology or the use of a	No/Very minor concerns<br><br>**Explanation:** The finding is rather descriptive. The data is coherent with the description of the finding. The finding reflects the whole of the data presented here.	Moderate concerns<br><br>**Explanation:** Moderate concerns regarding adequacy because we have only two contributing studies from two African countries but these provided relatively detailed data in relation to this descriptive finding. We note	Minor concerns<br><br>**Explanation:** Minor concerns regarding partial relevance because: - The healthcare settings: 1 of the studies matches the context of the review (NICU) , the other doesn't (but is in a surgery wards where HH is more likely to be	Moderate confidence<br><br>**Explanation:** Minor concerns regarding methodological limitations, No/Very minor concerns regarding coherence, Moderate concerns regarding adequacy, and Minor concerns regarding relevance We are concerned by the adequacy component and the lack of post-COVID, multi-setting data that would make us confident in the applicability of this finding across facilities and countries in	Mangochi et al. 2023; Yehouenou et al. 2022;

#	Summarised review finding	Methodological limitations	Coherence	Adequacy	Relevance	GRADE-CERQual assessment of confidence	References
		theoretical framework. However, we do not see these limitation deeply affecting this finding. We note that the researchers have taken into account the availability of training opportunities and have provided quotes that support the data (reducing the risk of individual bias on the value placed on training)		that the last part of the finding may be affected by how the training is offered or carried out (to all HCPs together or to one specific group at a time / facility-wide or ward-specific).	strictly applied). In addition, the studies are from two poorer African countries - We are unsure how training is implemented across different settings but we do not anticipate important differences in how HCWs value training.	Sub-Saharan Africa in today's world.	
3	Finding 3 – training for all staff: All staff (including HCWs, and support staff such as cleaners) believed that all range of staff should receive training on HH to contribute to infection	Minor concerns<br><br>**Explanation:** Minor concerns	No/Very minor concerns	Moderate concerns	Minor concerns<br><br>**Explanation:** Minor concerns	Moderate confidence<br><br>**Explanation:** Minor concerns regarding methodological	Mangochi et al. 2023; Yehouenou et al. 2022;

#	Summarised review finding	Methodological limitations	Coherence	Adequacy	Relevance	GRADE-CERQual assessment of confidence	References
	control. Cleaners and hospital attendants were seldom provided information and training opportunities and did not feel empowered to contribute to IPC. One cleaner also noted the importance for IPC of the training and awareness-raising that they had received.(2 studies)	regarding methodological limitations because of the lack of reflexivity. However, because of the descriptive nature of the finding (and the underlying data), we don't see this issue as a serious risk for the quality of methodological quality supporting this finding.	**Explanation:** The finding has a good fit with the data we have extracted. Ambiguity on which stakeholders are covered by the statement is better addressed under other criteria.	**Explanation:** Moderate concerns regarding adequacy because we only have 2 studies carried out before the COVID pandemic. The first part of the finding is expected to speak to the Sub-Saharan African context. We do note that COVID may have introduced some changes. We also can't be certain that the two settings represented in this finding would be representative of all NICUs on the	regarding relevance because we note that these 2 studies were conducted pre-COVID. The context of the data is relevant to the Sub-Saharan African setting. We note that changes have been observed in Sub-Saharan Africa further to COVID, with support staff (e.g. cleaning staff) being offered more training opportunities on HH, often from international organizations and wider WASH programs.	limitations, No/Very minor concerns regarding coherence, Moderate concerns regarding adequacy, and Minor concerns regarding relevance The lack of studies post COVID and how the situation has evolved since then raises questions as to how applicable the finding still is applicable to NICUs today. We would expect that the stronger focus on HH further to COVID may have affected the opportunities for training for all staff across the continent.	

#	Summarised review finding	Methodological limitations	Coherence	Adequacy	Relevance	GRADE-CERQual assessment of confidence	References
				continent. Due to the lack of data from studies carried out since the pandemic, the second part of the finding may no longer speak or be directly applicable to the situation in Sub-Saharan Africa today			
4	Finding 4 – reminders and posters: Most HCWs thought that reminders and posters to support HH compliance would be useful for HCWs. One nurse noted that these should be placed near all water sources. (1 study).	Minor concerns<br><br>**Explanation:** Minor concerns regarding methodological limitations because...The absence of reflexivity is unlikely to have affected the study.	No/Very minor concerns<br><br>**Explanation:** No data contradicting the finding. Descriptive study.	Minor concerns<br><br>**Explanation:** Minor concerns regarding adequacy because we only have one study from one African country but we do have 6 excerpts	Minor concerns<br><br>**Explanation:** Minor concerns regarding relevance because the study covers similar population as in a NICU ward across the continent. We expect that HH	Moderate confidence<br><br>**Explanation:** Minor concerns regarding methodological limitations, No/Very minor concerns regarding coherence, Minor concerns regarding adequacy, and Minor concerns regarding relevance Even though we don't have any major concerns, the data is very limited (1 study) which affect how	Yehouenou et al. 2022;

#	Summarised review finding	Methodological limitations	Coherence	Adequacy	Relevance	GRADE-CERQual assessment of confidence	References
		The issue related to the use of the analytical framework is most likely due to the reporting rather than a methodological flaw. The finding is descriptive and is unlikely to have been biased by these limitations		supporting this finding.	compliance in a surgery ward would be lower than in a NICU. However, we don't expect this would significantly change how HCP value reminders and poster or where they should be placed.	confident we can be in the finding in general	
5	Finding 5 –combining and incorporating posters into training: HCWs suggested that posters and other reminders should be implemented together with HH training, and thought that these together could have a considerable effect. (1 study)	No/Very minor concerns<br><br>**Explanation:** The limitations are unlikely to have biased or affected this finding in any significant way.	Minor concerns<br><br>**Explanation:** Minor concerns regarding coherence because the underlying data is ambiguous (refer to the extensive discussion we've	Moderate concerns<br><br>**Explanation:** Moderate concerns regarding adequacy because Only one study, 3 excerpts so comparatively	No/Very minor concerns<br><br>**Explanation:** This approach of combining posters and training is common across Sub-Saharan Africa. We don't expect the	Moderate confidence<br><br>**Explanation:** No/Very minor concerns regarding methodological limitations, Minor concerns regarding coherence, Moderate concerns regarding adequacy, and No/Very minor concerns regarding relevance. The data supporting this finding is very	Yehouenou et al. 2022;

#	Summarised review finding	Methodological limitations	Coherence	Adequacy	Relevance	GRADE-CERQual assessment of confidence	References
			had about what the data meant and how posters were used in this context). However, the study is descriptive and can be understood in context.	thinner data than for other findings	perception of HCP on this would be significantly different between Benin and other countries on the continent or between a surgery ward and a NICU.	limited (1 study, 3 excerpts). However, we do not expect significant variations or differences across Sub-Saharan African countries that would warrant further downgrading this finding.	
6	Finding 6 – role models and peer influence - to build a supportive institutional and management environment: HCWs noted the importance of role models to remind staff of hand hygiene guidelines and practices and helped improve adherence to guidelines. They also noted that peers in their team could help improve adherence by actively supporting hand hygiene practices and reminding colleagues to wash their hands. However, HCWs observed that some staff, including cleaners and hospital attendants, were seldom invited to ward team meetings and so	No/Very minor concerns<br><br>**Explanation:** The lack of reflexivity in the studies is unlikely to have affected the finding. The other limitations are most likely related to reporting and are unlikely to have significantly	No/Very minor concerns<br><br>**Explanation:** No ambiguity of the data, good fit between the underlying data and the finding.	Moderate concerns<br><br>**Explanation:** Moderate concerns regarding adequacy because we only have 2 studies with 4 and 2 excerpts respectively. This would provide quite thin data against the	Minor concerns<br><br>**Explanation:** Minor concerns regarding relevance because Role models (champions) are a strategy routinely promoted by international organization like the WHO. Therefore, it is common across	Low confidence<br><br>**Explanation:** No/Very minor concerns regarding methodological limitations, No/Very minor concerns regarding coherence, Moderate concerns regarding adequacy, and Minor concerns regarding relevance Limited number of studies, thin data in each study and also questions about whether part of the finding is directly applicable to all the Sub-Saharan	Mangochi et al. 2023; Yehouenou et al. 2022;

#	Summarised review finding	Methodological limitations	Coherence	Adequacy	Relevance	GRADE-CERQual assessment of confidence	References
	missed important information and training opportunities. This, they suggested, made these staff feel less empowered to contribute to IPC. (2 studies)	affected this finding. However, we do not that the broader institutional context may influence a researcher's approach to the issue and that this institutional context varies across Sub-Saharan Africa		variation in institutional and management environment found across Sub-Saharan Africa.	Sub-Saharan Africa. Champions supports the involvement of all types of staff in HH. Since COVID, there have been efforts across the continent to include all types of staff in hand hygiene. Therefore the second part of the finding might be less acute now days.	African contexts today (post-pandemic)	
7	Finding 7: Even when HCWs had a good knowledge of HHC guidelines, heavy workload combined with inadequate HH resources and infrastructure affected their capacity to implement good HH. HCW described how the lack of or inadequate	Minor concerns<br><br>**Explanation:** Minor concerns regarding methodological	No/Very minor concerns<br><br>**Explanation:** Descriptive findings that aligns	Moderate concerns<br><br>**Explanation:** Moderate concerns	Minor concerns<br><br>**Explanation:** Minor concerns regarding partial relevance because	Low confidence<br><br>**Explanation:** Minor concerns regarding methodological limitations, No/Very minor concerns regarding coherence,	Mangochi et al. 2023; Yehouenou et al. 2022;

#	Summarised review finding	Methodological limitations	Coherence	Adequacy	Relevance	GRADE-CERQual assessment of confidence	References
	infrastructure for HH, such as the lack of access to clean water or the poor placement of sinks, hampered good infection prevention and control. Poor HHC practice was further exacerbated when workload was heavy. (2 studies)	limitations because of limited descriptions of reflexivity in the studies. In one of the studies, the researchers were also healthcare workers and this could have influenced their perceptions of issues such as workload.	closely with the data from the primary studies.	regarding adequacy because we have only two contributing studies from two African countries but these provided relatively detailed data in relation to this descriptive finding.	the contributing studies come from two LICs where access to HH resources and levels of staffing may be lower than in other settings. The studies do however include relevant clinical settings and a range of healthcare workers and we anticipate that many African countries have hospitals that experience these constraints.	Moderate concerns regarding adequacy, and Minor concerns regarding relevance. This is because of the small number of studies from only two countries on the continent. However, this is a descriptive finding and we anticipate that many African countries have hospitals that experience similar constraints as those highlighted in these 2 studies.	

Appendix 11. Summary of Qualitative Findings for the Chatfield review

Tables adapted from: Chatfield, S. L., DeBois, K., Nolan, R., Crawford, H., & Hallam, J. S. (2017). Hand hygiene among healthcare workers: A qualitative meta summary using the GRADE-CERQual process. *Journal of infection prevention*, *18*(3), 104-120.

Findings that included studies from the African continent have been marked with a (*). The text marked in red in the Table indicates findings for which the GRADE-CERQual assessment has been adjusted in relation to use of the findings in the Sub-Saharan context.

Review finding	Overall confidence in finding	Explanation of GRADE-CERQual assessment	References
H1. HCW reported hand hygiene training is provided on a regular basis, and assert that HCW, as a group, are knowledgeable about hand hygiene practice. Some HCW recommended that changes to content or frequency of training might help improve the effectiveness of training, and extension of training to other staff, patients, families and community members might help improve compliance rates.*	Moderate	Most coded excerpts from studies with minimal/minor concerns about methods and moderate concerns about relevance; higher proportion of codes from studies with minimal concerns about coherence; minimal concerns with adequacy; 5/6 of studies represented in this finding	Barrett, Boscart, Brown, Bryce, Cole, Dawson, DeAbreu Botene, Dixit, Dyson, Ellingson, Erasmus, Eveillard, Jang (a), Jang (b), Jones, Joshi, Lee, Lusardi, Marjadi, McInnes, McLaws, Nichols, Nicol, Pan, Samuel, Siebert, Wasswa, White, Yuan, Zimmerman
H2 - HCW reported that their ability to engage in hand hygiene is influence by management-related factors that include availability of ample human and hygiene resources along with demonstrated support or priority on hand hygiene originating from upper levels of management. HCW also reported that it is important that hand hygiene improvement efforts solicit input from those 'in the trenches' to ensure their support.*	Moderate	Most coded excerpts from studies with minimal/minor concerns about methods and moderate concerns about relevance; coherence nearly evenly split between minimal concerns and substantial concerns; minimal concerns with adequacy; 5/6 of studies represented in this finding	Barrett, Boog, Boscart, Brown, Bryce, Cole, Dawson, Dyson, Ellingson, Erasmus, Eveillard, Jang (a), Jang (b), Jones, Joshi, Lusardi, Marjadi, McLaws, Nichols, Nicol, Pan, Patterson, Salmon, Samuel, Siebert, Wasswa, White, Yuan, Zimmerman

M2 - HCW reported that total compliance is not possible or practical given the realities of daily practice. HCW report actual and observed use of gloves as a time-saving alternative although often with less accompanying use of cleaners or sanitisers than is recommended; additionally, HCW reported that gloves are at times changed less frequently than recommended.*	Low	More contributing studies with minimal/minor concerns about methods vs. moderate/serious concerns; higher proportion of codes from studies with minimal to moderate concerns about coherence; minor concerns with adequacy; 2/3 of studies represented in this finding	Barrett, Boog, Cole, Dixit, Dyson, Eveillard, Hill, Jang (a), Jang (b), Joshi, Lee, Lusardi, Marjadi, McInnes, McLaws, Nichols, Pan, Patterson, Salmon, Samuel, Siebert, White, Zimmerman
L1. HCW reported that known surveillance is likely to improve compliance but indicated concerns with accuracy, communication and use of data gathered by electronic monitoring systems. However, there was not consensus among HCW in the sample regarding effectiveness of use of consequences (reward, punishment) in response to hand hygiene compliance.*	Very Low	More contributing studies with minimal/minor concerns about methods vs. moderate/serious concerns; half of studies represented; slightly more codes from studies with minimal vs. substantial concerns about coherence; very low proportion of total codes; mixed findings from the studies themselves	Boscart, Brown, Bryce, Dawson, Dyson, Ellingson, Jang (b), Johnson, Jones, Joshi, Lusardi, McInnes, McLaws, Pan, Salmon, Samuel, White, Yuan
L2. HCW reported that compliance with hand hygiene is a patient care practice that should be ubiquitous. HCW also reported that over time engaging in hand hygiene becomes an automatic behaviour.	Very Low	Half of studies represented; somewhat more codes from studies evidencing minimal to moderate concerns about coherence; very low proportion of total codes	Boscart, Brown, Cole, Dixit, Dyson, Eveillard, Hill, Jang (a), Jang (b), Johnson, Lusardi, McLaws, Nicol, Pan, Siebert, White, Yuan
L3. HCW reported that both hand hygiene behaviour and willingness to confront non-compliant others is influenced by hierarchy of staff within facilities. HCW reported that nurses are frequently perceived as beneath physicians; students reported that they perceived themselves as at risk if confronting non-compliant healthcare professionals.	Very Low	Half of studies represented; proportionately more codes from studies with moderate vs. substantial concerns about coherence; very low proportion of total codes	Barrett, Boscart, Brown, Bryce, Cole, Dixit, Erasmus, Jang (a), Jang (b), Lusardi, McInnes, McLaws, Nichols, Pan, Salmon, White, Yuan, Zimmerman
L4. HCW reported beliefs that are inconsistent with scientific evidence and are not necessarily swayed by presentation of evidence.	Very Low	Just over half of studies represented; somewhat more codes from studies with moderate vs. substantial concerns about coherence; very low proportion of total codes	Brown, Bryce, Cole, Dawson, DeAbreu Botene, Dixit, Dyson, Erasmus, Jang (b), Joshi, Marjadi, McInnes, Nichols, Nicol, Salmon, Siebert

Appendix 12. Qualitative Evidence Profile Table for the Chatfield review

The text marked in red in the Table indicates findings for which the GRADE-CERQual assessment has been adjusted in relation to use use of the findings in the Nigerian context.

Summarised review finding	References	Methodological limitations	Coherence	Adequacy	Relevance	GRADE-CERQual assessment of confidence
H1. HCW reported hand hygiene training is provided on a regular basis, and assert that HCW, as a group, are knowledgeable about hand hygiene practice. Some HCW recommended that changes to content or frequency of training might help improve the effectiveness of training, and extension of training to other staff, patients, families and community members might help improve compliance rates.	Barrett, Boscart, Brown, Bryce, Cole, Dawson, DeAbreu Botene, Dixit, Dyson, Ellingson, Erasmus, Eveillard, Jang (a), Jang (b), Jones, Joshi, Lee, Lusardi, Marjadi, McInnes, McLaws, Nichols, Nicol, Pan, Samuel, Siebert, Wasswa, White, Yuan, Zimmerman	minimal/minor concerns	minimal/minor concerns	minimal/minor concerns	moderate concerns	Moderate

H2. HCW reported that their ability to engage in hand hygiene is influence by management-related factors that include availability of ample human and hygiene resources along with demonstrated support or priority on hand hygiene originating from upper levels of management. HCW also reported that it is important that hand hygiene improvement efforts solicit input from those 'in the trenches' to ensure their support.	Barrett, Boog, Boscart, Brown, Bryce, Cole, Dawson, Dyson, Ellingson, Erasmus, Eveillard, Jang (a), Jang (b), Jones, Joshi, Lusardi, Marjadi, McLaws, Nichols, Nicol, Pan, Patterson, Salmon, Samuel, Siebert, Wasswa, White, Yuan, Zimmerman	minimal/minor concerns	moderate concerns	minimal concerns	moderate concerns	Moderate
M2. HCW reported that total compliance is not possible or practical given the realities of daily practice. HCW report actual and observed use of gloves as a time-saving alternative although often with less accompanying use of cleaners or sanitisers than is recommended; additionally, HCW reported that gloves are at times changed less frequently than recommended.	Barrett, Boog, Cole, Dixit, Dyson, Eveillard, Hill, Jang (a), Jang (b), Joshi, Lee, Lusardi, Marjadi, McInnes, McLaws, Nichols, Pan, Patterson, Salmon, Samuel, Siebert, White, Zimmerman	minimal/minor concerns	moderate concerns	Minor concerns	moderate concerns	Low
L1. HCW reported that known surveillance is likely to improve compliance but indicated concerns with accuracy, communication and use of data gathered by electronic monitoring systems. However, there was not consensus among HCW in the sample regarding effectiveness of use of consequences (reward, punishment) in response to hand hygiene compliance.	Boscart, Brown, Bryce, Dawson, Dyson, Ellingson, Jang (b), Johnson, Jones, Joshi, Lusardi, McInnes, McLaws, Pan, Salmon, Samuel, White, Yuan	minimal/minor concerns	minimal/minor concerns	moderate concerns	moderate concerns	Very Low
L2. HCW reported that compliance with hand hygiene is a patient care practice that should be ubiquitous. HCW also reported that over time engaging in hand hygiene becomes an automatic behaviour.	Boscart, Brown, Cole, Dixit, Dyson, Eveillard, Hill, Jang (a), Jang (b), Johnson, Lusardi, McLaws, Nicol, Pan, Siebert, White, Yuan	minimal/minor concerns	minimal to moderate concerns	Moderate concerns	moderate concerns	Very Low
L3. HCW reported that both hand hygiene behaviour and willingness to confront non-compliant others is influenced by hierarchy of staff within facilities. HCW reported that nurses are frequently perceived as beneath physicians; students reported that they perceived themselves as at risk if confronting non-compliant healthcare professionals.	Barrett, Boscart, Brown, Bryce, Cole, Dixit, Erasmus, Jang (a), Jang (b), Lusardi, McInnes, McLaws, Nichols, Pan, Salmon, White, Yuan, Zimmerman	minimal/minor concerns	moderate vs. substantial concerns	**Substantial concerns**	Substantial concerns	Very Low

L4. HCW reported beliefs that are inconsistent with scientific evidence and are not necessarily swayed by presentation of evidence.	Brown, Bryce, Cole, Dawson, DeAbreu Botene, Dixit, Dyson, Erasmus, Jang (b), Joshi, Marjadi, McInnes, Nichols, Nicol, Salmon, Siebert	minimal/minor concerns	moderate vs. substantial concerns	**Substantial concerns**	moderate concerns	Very Low

Table adapted from: Chatfield, S. L., DeBois, K., Nolan, R., Crawford, H., & Hallam, J. S. (2017). Hand hygiene among healthcare workers: A qualitative meta summary using the GRADE-CERQual process. *Journal of infection prevention*, *18*(3), 104-120.

Appendix 13: Confidence assessments in the synthesised findings

WHO domain for hand hygiene compliance	Synthesised finding	Contributing findings – Chatfield review	GRADE-CERQual assessment of the Chatfield et al. finding	Contributing findings – de novo QES	GRADE-CERQual assessment of the de novo finding	GRADE-CERQual assessment of the synthesised finding
Acceptability of training among healthcare workers	**Finding 1**<br>While some HCWs noted that HHC becomes an automatic behaviour over time, others reported being unsure if their HH practice was correct. In studies from African settings, healthcare professionals and cleaning staff acknowledged the role of training in HH practices. They wanted more training, delivered frequently, that included locum staff and that covered theoretical and practical aspects of low confidence finding HH, including training in HH techniques. Some HCWs also felt that HH training could provide opportunities to discuss HH best practice. HCWs from other settings outside SSA, however, suggested that HH practice is natural and should be intrinsically motivated but acknowledged that this was not always the case	**Chatfield L2**<br>HCW reported that compliance with hand hygiene is a patient care practice that should be ubiquitous. HCW also reported that over time engaging in hand hygiene becomes an automatic behaviour. (17 studies, none from Sub-Saharan Africa)	Initial assessment by Chatfield et al. was **low** confidence due to minimal to moderate concerns over the coherence domain and moderate concerns over the adequacy domain.<br><br>All but four studies were from the Global North, in a variety of facility types (the type of ward was not provided). The lack of studies from settings comparable to NICU settings in Sub-Saharan Africa led us to downgrade the relevance component, leading to a downgrading of the overall assessment from low to **very low**.	**De novo QES – Finding 1**<br>Some HCWs reported being unsure that their HH practice was correct. Healthcare professionals and cleaning staff wanted more training, delivered frequently and that included theoretical and practical aspects of HH. They particularly noted that training in HH technique was lacking. Some nurses also noted that locum nurses did not always get sufficient orientation on IPC (2 studies)	**Moderate confidence**<br>*Minor concerns regarding methodological limitations, Moderate concerns regarding coherence, Moderate concerns regarding adequacy, and Minor concerns regarding relevance.*<br>There is uncertainty due to the limited number of studies and the ambiguity of some of the data or imprecision in the information available. Due to the low number of studies included, we cannot be certain that this finding would be applicable to all NICU settings in Sub-Saharan Africa, although feedback from practitioners tells us that variations across countries for this level and type of care might be limited. We do note that this finding is based on	**Low confidence finding**<br>We had moderate concerns about coherence as the contributing findings are not fully coherent with one another, even though the merged finding is mainly descriptive<br>The TRANSFER meeting supported some elements of the findings but not all of them. Therefore, it is unclear whether the synthesised finding is a reasonable representation of the views of HCWs across NICUs in Sub-Saharan Africa.<br><br>**We chose a conservative assessment of our confidence to reflect our concerns over coherence and uncertainty about the relevance of the finding**

					studies carried out pre-COVID, which raises uncertainty as to how applicable it is today.	**for a NICU in Sub-Saharan Africa today.**
				**De novo QES - Finding 2**<br>HCWs valued training and education for HH compliance. They acknowledged and knew the role of training in good HH practices and noted the need for more training. Some HCWs also felt that HH training could be an opportunity for discussions on HH best practices. (2 studies)	**Moderate confidence**<br>*Minor concerns regarding methodological limitations, No/Very minor concerns regarding coherence, Moderate concerns regarding adequacy, and Minor concerns regarding relevance.*<br>We are concerned by the adequacy component and the lack of post-COVID, multi-setting data that would make us confident in the applicability of this finding across facilities and countries in Sub-Saharan Africa in today's world.	

	**Finding 2**<br>Some studies noted that adequate HH training for HCWs was available but that the content and reach of training could be enhanced. Other studies suggested that all cadres should receive training on HH. They noted in particular that cleaners and hospital attendants were seldom provided information and training opportunities and did not feel empowered to contribute to IPC. Some HCWs noted that non-clinical staff were seldom invited to ward team meetings and so missed important information and training opportunities.<br>HCWs suggested that training directed at those individuals could potentially help reduce healthcare-acquired infection rates.	**Chatfield H1**<br>HCW reported hand hygiene training is provided on a regular basis, and assert that HCW, as a group, are knowledgeable about hand hygiene practice. Some HCW recommended that changes to content or frequency of training might help improve the effectiveness of training, and extension of training to other staff, patients, families and community members might help improve compliance rates. (31 studies including 3 from Sub-Saharan Africa)	Initial assessment by Chatfield et al. was **high** confidence – minimal or minor concerns across all domains.<br><br>The low number of studies from Sub-Saharan Africa, the diversity of facility types covered, as well as the lack of details on the type of ward and care setting led us to downgrade the relevance component, leading to a downgrading of the overall assessment from high to **moderate**.	**De novo QES - Finding 3**<br>All staff (including HCWs, and support staff such as cleaners) believed that all range of staff should receive training on HH to contribute to infection control. Cleaners and hospital attendants were seldom provided information and training opportunities and did not feel empowered to contribute to IPC. One cleaner also noted the importance for IPC of the training and awareness-raising that they had received. (2 studies)	**Moderate confidence**<br>*Minor concerns regarding methodological limitations, No/Very minor concerns regarding coherence, Moderate concerns regarding adequacy, and Minor concerns regarding relevance.*<br>The lack of studies post COVID and how the situation has evolved since then raises questions as to how applicable the finding still is applicable to NICUs today. We would expect that the stronger focus on HH further to COVID may have affected the opportunities for training for all staff across the continent.	**Low confidence finding**<br>We had no / very minor concerns about coherence as the three contributing findings are coherent with one another. However, stakeholders did highlight recent changes in HH practices in the region further to the COVID-19 pandemic, that are not reflected in the contributing findings. We therefore decided to further downgrade the synthesised finding to reflect relevance concerns about whether it is applicable to all NICUs in Sub-Saharan Africa today.<br><br>**We chose a conservative assessment of our confidence to reflect concerns about the relevance of the finding for the region in the post-COVID era.**
				**De novo QES - Finding 6 –** HCWs noted the importance of role models to remind staff of hand hygiene guidelines and practices and help improve adherence to guidelines. They also noted that peers in their team could help improve	**Low confidence**<br>*No/Very minor concerns regarding methodological limitations, No/Very minor concerns regarding coherence, Moderate concerns regarding adequacy, and*	

				adherence by actively supporting hand hygiene practices and reminding colleagues to wash their hands. However, HCWs observed that some staff, including cleaners and hospital attendants, were seldom invited to ward team meetings and so missed important information and training opportunities. This, they suggested, made these staff feel less empowered to contribute to IPC. (2 studies)	*Minor concerns regarding relevance.* Limited number of studies, thin data in each study and also questions about whether part of the finding is directly applicable to all the Sub-Saharan African contexts today (post-pandemic)	
Acceptability of reminders and communication among healthcare workers	**Finding 3** Most HCWs thought that reminders and posters to support HH compliance would be useful for HCWs. One nurse noted that these should be placed near all water sources. HCWs also suggested that posters and other reminders should be implemented together with HH training, and thought that these together could have a considerable effect.	**N/A**	**N/A**	**De novo QES - Finding** 4 Most HCWs thought that reminders and posters to support HH compliance would be useful for HCWs. One nurse noted that these should be placed near all water sources. (1 study).	**Moderate confidence** *Minor concerns regarding methodological limitations, No/Very minor concerns regarding coherence, Minor concerns regarding adequacy, and Minor concerns regarding relevance.* Even though we don't have any major concerns, the data is very limited (1 study) which affects how confident we can be in the finding in general.	**Moderate confidence** We had very minor/minor concerns about coherence, reflecting our assessment of the contributing findings. However, the contributing findings are affected by the limited underlying data, thus affecting the adequacy of the synthesised finding. However, the synthetised finding is descriptive and data from the TRANSFER meeting

				**De novo QES - Finding** 5<br>HCWs suggested that posters and other reminders should be implemented together with HH training, and thought that these together could have a considerable effect. (1 study)	**Moderate confidence**<br>*No/Very minor concerns regarding methodological limitations, Minor concerns regarding coherence, Moderate concerns regarding adequacy, and No/Very minor concerns regarding relevance.*<br>The data supporting this finding is very limited (1 study, 3 excerpts).	suggest that this finding would be relevant to different types of wards and countries in Sub-Saharan Africa.<br><br>**We therefore chose to maintain the assessments from the contributing findings.**

Acceptability of reminders and communication among healthcare workers	**Finding 4**<br>Healthcare professionals highlighted the importance of support from higher ranks in the organisation and a supportive institutional environment for HHC, allowing role models and senior staff to remind their colleagues to follow good HH practices.<br><br>However, healthcare professionals also noted that a hierarchical environment may make this type of intervention less effective if more junior or non-clinical staff do not feel empowered to be role models or to remind more senior staff	**Chatfield L3.**<br>HCW reported that both hand hygiene behaviour and willingness to confront non-compliant others are influenced by the hierarchy of staff within facilities. HCW reported that nurses are frequently perceived as beneath physicians; students reported that they perceived themselves as at risk if confronting non-compliant healthcare professionals.<br>(Low confidence findings)	Initial assessment by Chatfield et al. was **Low** confidence due to substantial concerns over the adequacy domain.<br><br>All but four studies were from the Global North, in different types of hospitals (ward unknown). The lack of studies from settings comparable to NICUs in Sub-Saharan Africa led us to downgrade the relevance component, leading to a downgrading of the overall assessment from low to very low	**De novo QES - Finding 6 –** HCWs noted the importance of role models to remind staff of hand hygiene guidelines and practices and help improve adherence to guidelines. They also noted that peers in their team could help improve adherence by actively supporting hand hygiene practices and reminding colleagues to wash their hands. However, HCWs observed that some staff, including cleaners and hospital attendants, were seldom invited to ward team meetings and so missed important information and training opportunities. This, they suggested, made these staff feel less empowered to contribute to IPC. (2 studies)	**Low confidence**<br>*No/Very minor concerns regarding methodological limitations, No/Very minor concerns regarding coherence, Moderate concerns regarding adequacy, and Minor concerns regarding relevance.*<br>Limited number of studies, thin data in each study and also questions about whether part of the finding is directly applicable to all the Sub-Saharan African contexts today (post-pandemic)	**Low confidence**<br>We had no / very minor concerns about coherence as the contributing findings are coherent with one another, both highlight the same factor (role of support from the hierarchy) and the synthesised finding is descriptive<br>The role of management and hierarchy did not come up in the TRANSFER meeting. Therefore, we relied on the limited data from the two QES (affecting adequacy) and had concerns about relevance to the diverse intensive care contexts in Sub-Saharan Africa.<br><br>**We chose a conservative assessment of our confidence to reflect concerns about the relevance of the finding for Sub-Saharan Africa.**

Acceptability of monitoring and feedback among healthcare workers	**Finding 5**<br>The perceived need for and acceptability of monitoring and surveillance interventions for HHC varied across studies. In some studies, HCWs acknowledged that being aware of surveillance, whether via direct monitoring from staff or electronic monitoring (e.g., use of video cameras), might improve HH practice. However, in studies conducted outside of SSA, other HCWs questioned whether monitoring was actually needed as these practices should be an	**Chatfield L.1**<br>HCW reported that known surveillance is likely to improve compliance but indicated concerns with accuracy, communication and use of data gathered by electronic monitoring systems. However, there was no consensus among HCW in the sample regarding effectiveness of use of consequences (reward, punishment) in response to hand hygiene compliance. (18 studies, including 2 from Sub-Saharan Africa)	Initial assessment by Chatfield et al. was **low** confidence due to due to moderate concerns about the adequacy domain.<br><br>The low number of studies from Sub-Saharan Africa as well as the lack of details on the type of ward and care setting led us to downgrade the relevance component, leading to a downgrading of the overall assessment from low to **very low**.	**N/A**	**N/A**	**Very low confidence**<br>Only findings from the Chatfield et al. review contributed to this synthesised finding. The confidence assessments in these findings had indicated some concerns over adequacy and, for one contributing finding, coherence.<br>Further, the TRANSFER meeting did not provide much detail on surveillance interventions in Sub-Saharan Africa as stakeholders had limited

	instinctive part of care and would become an automatic behaviour with time and experience.	**Chatfield L2**<br>HCW reported that compliance with hand hygiene is a patient care practice that should be ubiquitous. HCW also reported that over time engaging in hand hygiene becomes an automatic behaviour. (17 studies, none from Sub-Saharan Africa)	Initial assessment by Chatfield et al. was **low** confidence due to minimal to moderate concerns over the coherence domain and moderate concerns over the adequacy domain.<br><br>All but four studies were from the Global North, in a variety of facility types (ward unknown). The lack of studies from settings comparable to NICU settings in Sub-Saharan Africa led us to downgrade the relevance component, leading to a downgrading of the overall assessment from low to **very low**.			experience with this type of intervention.<br>These concerns about adequacy (the lack of data from the region) and the relevance of the finding to the diverse intensive care contexts in Sub-Saharan Africa reduced our confidence in the finding.<br><br>**We chose to maintain of our revised very low confidence assessment to reflect our concerns about adequacy and uncertainty about the relevance of the finding for Sub-Saharan Africa.**

	**Finding 6**<br>In studies conducted outside of SSA, HCWs noted that monitoring data should be communicated in a timely manner and in sufficient detail to be useful. They explained that HAI data collected in facilities were not useful when not made available in a timely way or not provided with ample detail. HCWs could lose confidence in data when these lacked contextual information.	**Chatfield L4**<br>HCW reported beliefs that are inconsistent with scientific evidence and are not necessarily swayed by presentation of evidence. (16 studies, none from Sub-Saharan Africa)	Initial assessment by Chatfield et al. was **low** confidence due to substantial concerns over the adequacy domain and moderate to substantial concerns over the coherence domains.<br><br>All but four studies were from the Global North, in a variety of facility types (ward unknown). The lack of studies from Sub-Saharan Africa, the diversity of facility types covered, as well as the lack of details on the type of ward and care setting led us to downgrade the relevance component, leading to a downgrading of the overall assessment from low to **very low**.	**N/A**	**N/A**	**Very low confidence**<br>Only one finding from the Chatfield et al. review contributed to this synthesised finding. Further, the TRANSFER meeting participants supported the general usefulness of timely monitoring data.<br>We therefore had concerns related to adequacy due to the lack of data from the region and concerns about the relevance of the finding to diverse intensive care contexts in Sub-Saharan Africa is unknown.<br><br>**We chose to maintain our very low confidence assessment to reflect our concerns about adequacy and uncertainty about the relevance of the finding for Sub-Saharan Africa.**

Feasibility of HHC interventions	**Finding 7**<br>Management support, demonstrated by the provision of sufficient human and hygiene resources, was necessary but frequently lacking thus hampering good IPC practice.<br>HCWs noted that asking those involved directly in providing care for their views encouraged 'buy-in' from lower levels of the organizational structure.	**Chatfield H2**<br>HCW reported that their ability to engage in hand hygiene is influence by management-related factors that include availability of ample human and hygiene resources along with demonstrated support or priority on hand hygiene originating from upper levels of management. HCW also reported that it is important that hand hygiene improvement efforts solicit input from those 'in the trenches' to ensure their support. (29 studies, including 3 studies in Sub-Saharan Africa)	Initial assessment by Chatfield et al. was **high** (minimal concerns across all domains but coherence, which had moderate concerns).<br><br>The low number of studies from Sub-Saharan Africa, the diversity of facility types covered, as well as the lack of details on the type of ward and care setting led us to downgrade the relevance component, leading to a downgrading of the overall assessment from high to **moderate**.	**De novo QES - Finding 7**<br>Even when HCWs had a good knowledge of HHC guidelines, heavy workload combined with inadequate HH resources and infrastructure affected their capacity to implement good HH. HCW described how the lack of or inadequate infrastructure for HH, such as the lack of access to clean water or the poor placement of sinks, hampered good infection prevention and control. Poor HHC practice was further exacerbated when the workload was heavy. (2 studies)	**Low confidence**<br>*Minor concerns regarding methodological limitations, No/Very minor concerns regarding coherence, Moderate concerns regarding adequacy, and Minor concerns regarding relevance.*<br>Only two studies contributing to this finding were from two African countries, but these provided relatively detailed data in relation to this descriptive finding.<br>The contributing studies come from two low-income countries where access to HH resources and levels of staffing may be lower than in other settings.<br>Limited descriptions of reflexivity in the studies.	**Low confidence**<br>We had no / very minor concerns about coherence as the two contributing findings are aligned with one another and the merged finding is mainly descriptive. The TRANSFER meeting did not address the issue of management support but did highlight the issue of human and hygiene resources. We therefore had adequacy concerns related to the limited amount of data available from the region, and concerns about whether the finding would be directly relevant to the diverse intensive care contexts in Sub-Saharan Africa.<br><br>**We chose a conservative assessment of our confidence to reflect uncertainty about the relevance of the finding for Sub-Saharan Africa.**

	**Finding 8**<br>Even though HCWs might know what good IPC practices were, workload affected their ability to implement these. HCWs noted that they did not have enough time to fully comply with HH policies while managing patient care. Furthermore, some HCWs reported observing that gloves were being used as an alternative to HH practice when facing a heavy workload.<br>Evidence from outside SSA also suggested that HCWs questioned the evidence base linking HH and HAI and the value of recommended HH practices.	**Chatfield L4**<br>HCW reported beliefs that are inconsistent with scientific evidence and are not necessarily swayed by the presentation of evidence. (16 studies, none from Sub-Saharan Africa)	Initial assessment by Chatfield et al. was **low** confidence due to substantial concerns over the adequacy domain and moderate to substantial concerns over the coherence domains.<br><br>All but four studies were from the Global North, in a variety of facility types (the type of ward was not provided). The lack of studies from Sub-Saharan Africa, the diversity of facility types covered, as well as the lack of details on the type of ward and care setting led us to downgrade the relevance component, leading to a downgrading of the overall assessment from low to **very low**.	**De novo QES - Finding 7**<br>Even when HCWs had a good knowledge of HHC guidelines, heavy workload combined with inadequate HH resources and infrastructure affected their capacity to implement good HH. HCW described how the lack of, or inadequate infrastructure for HH, such as the lack of access to clean water or the poor placement of sinks, hampered good infection prevention and control. Poor HHC practice was further exacerbated when the workload was heavy. (2 studies)	**Low confidence**<br>*Minor concerns regarding methodological limitations, No/Very minor concerns regarding coherence, Moderate concerns regarding adequacy, and Minor concerns regarding relevance.*<br>Only two studies contributing to this finding were from two African countries, but these provided relatively detailed data in relation to this descriptive finding.<br>The contributing studies come from two low-income countries where access to HH resources and levels of staffing may be lower than in other settings.<br>Limited descriptions of reflexivity in the studies.	**Low confidence**<br>The finding from the de novo QES was the main contributor to this synthesised finding. We had concerns about adequacy as the de novo finding was based on only 2 studies. Further, the TRANSFER meeting did not mention healthcare providers' attitudes towards scientific evidence but did address workload as a common barrier.<br>The data from Sub-Saharan Africa comes exclusively from the de novo QES finding.<br><br>**We chose to apply the assessment from the de novo QES finding to reflect the greater contribution of this finding to the synthesised finding, the comparatively limited**

		**Chatfield M2**<br>HCW reported that total compliance is not possible or practical given the realities of daily practice. HCW report actual and observed use of gloves as a time-saving alternative although often with less accompanying use of cleaners or sanitisers than is recommended; additionally, HCW reported that gloves are at times changed less frequently than recommended. (23 studies, including 1 study from Sub-Saharan Africa)	Initial assessment by Chatfield et al. was moderate due to moderate concerns over the coherence domain.<br><br>All but five studies were from the Global North, in a variety of facility types (ward unknown). The lack of studies from Sub-Saharan Africa, the diversity of facility types covered, as well as the lack of details on the type of ward and care setting led us to downgrade the relevance component, leading to a downgrading of the overall assessment from moderate to **low**.			**data (adequacy) and uncertainty about the relevance of the finding for the whole of Sub-Saharan Africa.**
